# Increased PLEKHO1 within osteoblasts suppresses Smad‐dependent BMP signaling to inhibit bone formation during aging

**DOI:** 10.1111/acel.12566

**Published:** 2017-01-13

**Authors:** Jin Liu, Chao Liang, Baosheng Guo, Xiaohao Wu, Defang Li, Zongkang Zhang, Kang Zheng, Lei Dang, Xiaojuan He, Changwei Lu, Songlin Peng, Xiaohua Pan, Bao‐Ting Zhang, Aiping Lu, Ge Zhang

**Affiliations:** ^1^ Institute for Advancing Translational Medicine in Bone & Joint DiseasesSchool of Chinese MedicineHong Kong Baptist UniversityHong Kong SARChina; ^2^ Institute of Integrated Bioinfomedicine and Translational ScienceSchool of Chinese MedicineHong Kong Baptist UniversityHong Kong SARChina; ^3^ Institute of Precision Medicine and Innovative Drug DiscoveryHong Kong Baptist UniversityHong Kong SARChina; ^4^ School of Chinese MedicineFaculty of MedicineThe Chinese University of Hong KongHong Kong SARChina; ^5^ Institute of Basic Research in Clinical MedicineChina Academy of Chinese Medical SciencesBeijingChina; ^6^ Department of OrthopaedicsXi'an Third HospitalXi'an, ChinajingChina; ^7^ Department of Spine SurgeryShenzhen People's HospitalJi Nan University Second College of MedicineShenzhenChina; ^8^ Department of Orthopaedics and TraumatologyBao'an Hospital Affiliated to Southern Medical University & Shenzhen 8th People HospitalShenzhenChina

**Keywords:** aging, BMP signaling, osteoblast, osteoporosis

## Abstract

Emerging evidence indicates that the dysregulation of protein ubiquitination plays a crucial role in aging‐associated diseases. Smad‐dependent canonical BMP signaling pathway is indispensable for osteoblastic bone formation, which could be disrupted by the ubiquitination and subsequent proteasomal degradation of Smad1/5, the key molecules for BMP signaling transduction. However, whether the dysregulation of Smad1/5 ubiquitination and disrupted BMP signaling pathway is responsible for the age‐related bone formation reduction is still underexplored. Pleckstrin homology domain‐containing family O member 1 (PLEKHO1) is a previously identified ubiquitination‐related molecule that could specifically target the linker region between the WW domains of Smurf1 to promote the ubiquitination of Smad1/5. Here, we found an age‐related increase in the expression of PLEKHO1 in bone specimens from either fractured patients or aging rodents, which was associated with the age‐related reduction in Smad‐dependent BMP signaling and bone formation. By genetic approach, we demonstrated that loss of *Plekho1* in osteoblasts could promote the Smad‐dependent BMP signaling and alleviated the age‐related bone formation reduction. In addition, osteoblast‐specific *Smad1* overexpression had beneficial effect on bone formation during aging, which could be counteracted after overexpressing *Plekho1* within osteoblasts. By pharmacological approach, we showed that osteoblast‐targeted *Plekho1* siRNA treatment could enhance Smad‐dependent BMP signaling and promote bone formation in aging rodents. Taken together, it suggests that the increased PLEKHO1 could suppress Smad‐dependent BMP signaling to inhibit bone formation during aging, indicating the translational potential of targeting PLEKHO1 in osteoblast as a novel bone anabolic strategy for reversing established osteoporosis during aging.

## Introduction

Aging‐induced reduction in osteoblastic bone formation results in decreased bone mass and deteriorated bone architecture, leading to increased fracture risk in elderly patients (Boskey & Coleman, 2010). However, the underlying molecular mechanism responsible for the reduced bone formation during aging remains largely unknown. Albeit emerging studies have established that the ubiquitin/proteasome system is indispensable for osteoblastic bone formation (Zhao *et al*., 2004; Yamashita *et al*., 2005; Kim *et al*., 2011; Qiang *et al*., 2012; Severe *et al*., 2013; Zhang & Xing, 2012; Choi *et al*., 2015; Tsubakihara *et al*., 2015), the extent to which it contributes to the aging‐induced bone formation reduction is underexplored.

The bone morphogenetic protein (BMP) signaling pathway is one of the key signaling pathways responsible for osteoblastic bone formation, wherein Smad1 and Smad5 are the key mediators involved in the signal transduction of canonical BMP pathway and play a crucial role in regulating osteoblastic bone formation (Retting *et al*., 2009; Song *et al*., 2009; Wang *et al*., 2011). The ubiquitination and subsequent proteasomal degradation of Smad1/5 represent one of the crucial regulatory processes for controlling the BMP signaling cascade, which is mediated by the HECT family of E3 ubiquitin ligases, *for example,* Smad ubiquitin regulatory factor (Smurf) including Smurf1 and Smurf2 (Zhang *et al*., 2013; Yamashita *et al*., 2005; Xing *et al*., 2010). Pleckstrin homology domain‐containing family O member 1 (PLEKHO1, also known as CKIP‐1) is a previously identified ubiquitination‐related molecule that could negatively regulate BMP signaling pathway in HEK293T cells, in which PLEKHO1 could facilitate Smurf1‐mediated ubiquitylation of Smad1/5 *in vitro* (Lu *et al*., 2008). Although systemic depletion of *Plekho1* gene in mice could result in high bone mass (Lu *et al*., 2008), several recent studies further demonstrated that PLEKHO1 was involved in regulating adipogenesis (Li *et al*., 2009), cardiomyocyte hypertrophy (Ling *et al*., 2012), and inflammation responses (Juhasz *et al*., 2013). Thus, the systemic *Plekho1* knockout mice may not be the ideal genetic mouse model to provide precise understandings on how PLEKHO1 regulates bone formation in cell‐specific manner. Moreover, it remains underdetermined whether PLEKHO1 regulates the Smad‐dependent canonical BMP signaling in osteoblasts and how this regulatory mechanism contributes to the age‐related bone formation reduction.

In this study, we found an age‐related increase in PLEKHO1 expression in bone specimens from either fractured patients or aging rodents, which was associated with the age‐related reduction in Smad‐dependent BMP signaling and bone formation. By genetic approach, we demonstrated that loss of *Plekho1* in osteoblasts could promote the Smad‐dependent canonical BMP signaling and alleviated the age‐related bone formation reduction. In addition, osteoblast‐specific *Smad1* overexpression had beneficial effect on bone formation during aging, which could be counteracted after overexpressing *Plekho1* within osteoblasts. By pharmacological approach, we showed that osteoblast‐targeted *Plekho1* siRNA treatment could enhance Smad‐dependent BMP signaling and promote bone formation in aging rodents.

## Results

### High PLEKHO1 in osteoblasts accompanied by the decreased Smad‐dependent BMP signaling and reduced bone formation during aging

**Figure 1:**
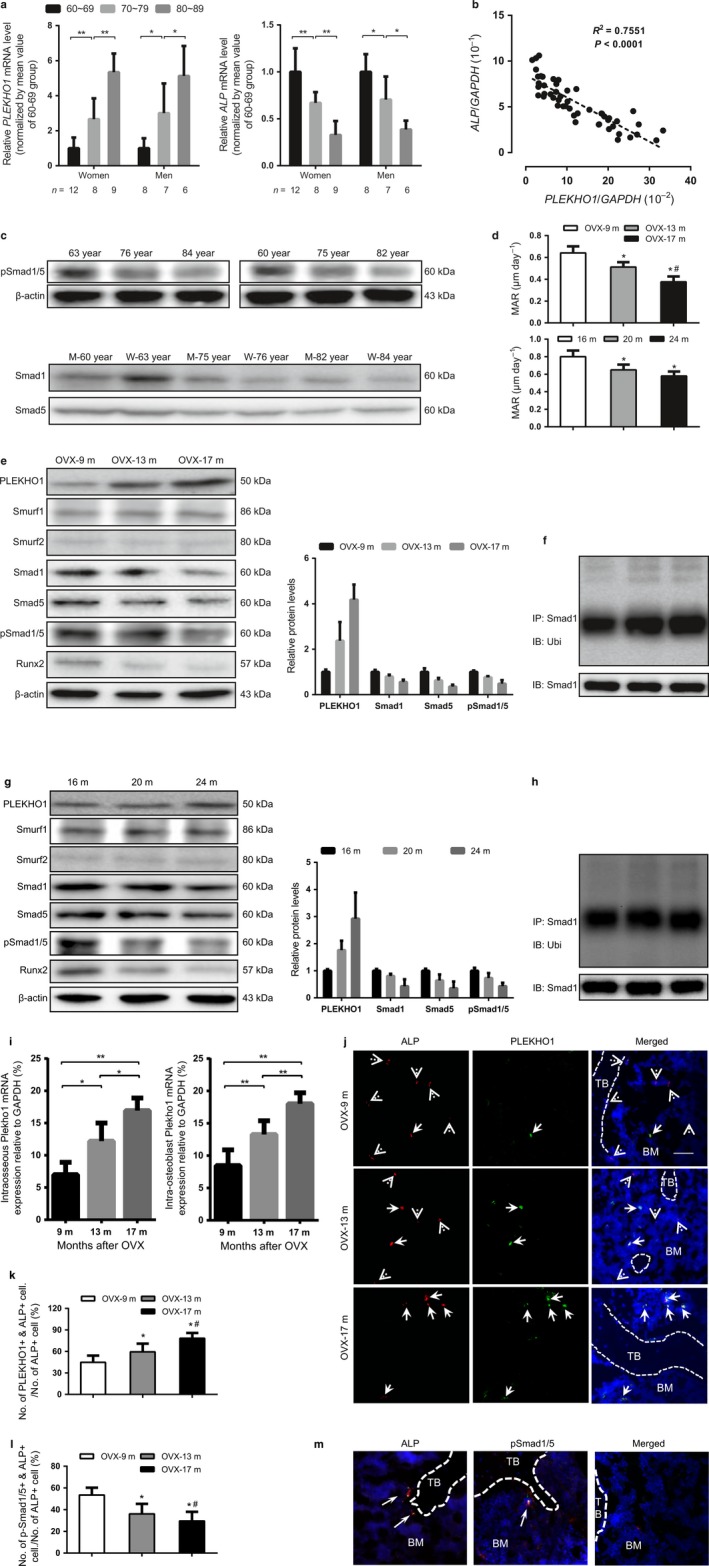
Increased expression of PLEKHO1 within osteoblast accompanied by reduced Smad1‐dependent BMP signaling and decreased bone formation during aging. (a) The age‐related changes of *PLEKHO1* (left) and *ALP* (right) mRNA levels in bone specimens from women and men with fracture, respectively. (b) Correlation analysis between *PLEKHO1* mRNA level and *ALP* mRNA level in bone specimens from fractured patients. (c) Representative electrophoretic bands of the phosphorylated Smad1/5 (pSmad1/5) and total Smad1/5 proteins in bone specimens from women and men with fracture, respectively. M: men, W: women, yr: years of age. (d) The quantitative data of mineral apposition rate (MAR) at distal femur from aging ovariectomized (OVX) rats at 9 (OVX‐9m), 13 (OVX‐13m), and 17 (OVX‐17m) months after OVX (top) and male rats at 16, 20, and 24 months of age (bottom). (e, g) Western blot analysis of the protein levels of PLEKHO1, Smurf‐1, Smurf2, Smad1, Smad5, and pSmad1/5 in tibias from aging OVX rats (upper panel) and male rats (lower panel) at the indicated time points. Left: The representative electrophoretic bands. Left: The semiquantitative data of the protein levels. (f, h) The representative electrophoretic bands for the ubiquitination levels of Smad1 in 2nd ~ 4th lumbar vertebrates (LV 2–4) from aging OVX rats (upper panel) and male rats (lower panel) at the indicated time points. The rats were treated with MG132 (2 mg kg^−1^) through intraperitoneal injection 24 h before sample collections. (i) The age‐related changes of *Plekho1* mRNA levels in whole bone tissue (left) and osteoblast (right) from aging OVX rats. (j) The quantitative data of the ratio of PLEKHO1 and ALP co‐positive cells (PLEKHO1+ & ALP+) among the ALP+ cells at distal femur from aging OVX rats at the indicated time points. (k) The representative fluorescence micrographs of the protein expression of PLEKHO1 (green) in ALP+ (red) cells at distal femora from aging OVX rats at the indicated time points. Scale bar = 50 μm. Dotted arrow indicated the cells expressing ALP but not PLEKHO1; solid arrow indicates the cells co‐expressing pSmad1/5 and ALP. Dotted line indicates bone surface. BM: bone marrow; TB: trabecular bone. (l) The quantitative data of the ratio of pSmad1/5 and ALP co‐positive cells (pSmad1/5+ & ALP+) among the ALP+ cells at distal femur from aging OVX rats at the indicated time points. (m) The representative fluorescence micrographs of the protein expression of pSmad1/5 (green) in ALP+ (red) cells from aging OVX rats at the indicated time points. Scale bar = 50 μm. Arrow indicates the cells co‐expressing p‐Smad1/5 and ALP. *Note:* All data are the mean ± SD. In the study with human specimens, the relative mRNA levels are normalized to the mean value of the 60–69 years group. Human *GAPDH* mRNA and β‐actin protein are used as the internal controls. *P < 0.05, **P < 0.01. In the study with rat specimens, the relative mRNA levels and protein levels are normalized to the mean value of the OVX‐9m or 16m group. Rat *Gapdh* mRNA and β‐actin protein are used as the internal controls. In (d), (k), & (m), *P < 0.05 when compared to the 9 months after OVX group. #*P* < 0.05 when compared to the 13 months after OVX group. In (i), *P < 0.05, **P < 0.01. One‐way analysis of variance (ANOVA) with a *post hoc* test was performed.

We collected bone specimens from 50 elderly patients (29 women and 21 men, 60–89 years old) with fracture in three clinical centers (Table S1, Supporting information), wherein we observed a significant age‐related increase in the mRNA levels of *PLEKHO1* in those bone samples from both elderly women and men with fracture, while the mRNA levels of *ALP* (alkaline phosphatase, a bone formation marker gene) decreased with age (Fig. 1a). The *PLEKHO1* mRNA expression level was negatively correlated with the *ALP* mRNA expression level during aging (Fig. 1b). Furthermore, Western blot analysis showed an age‐related decrease in the protein levels of phosphorylated Smad1/5 (p‐Smad1/5) and total Smad1/5 (Fig. 1c).

Given that PLEKHO1 is highly conserved between human and rodents, we next investigated the expression pattern of PLEKHO1 and the alteration of BMP signaling at both tissue (bone) and cellular (osteoblast) levels in two rodent models with age‐related bone loss, *that is,* aging ovariectomized (OVX) rats and aging male rats. The female rats were ovariectomized at 4 months of age and sacrificed at 9 (OVX‐9m), 13 (OVX‐13m), and 17 months (OVX‐17m) after OVX, that is, 13, 17, and 21 months of age, to mimic the age‐related bone formation reduction in the aging postmenopausal women (Li *et al*., 2014). The male rats were sacrificed at 16 (16m), 20 (20m), and 24 months (24m) of age to mimic the age‐related bone formation reduction in the aging men. Micro‐CT analysis showed that the bone mineral density (BMD) and ratio of bone volume to tissue volume (BV/TV) both decreased in the sham‐operated female rats during aging, while the above age‐related decreases were accelerated after OVX (Fig. S1a, Supporting information). Bone histomorphometry analysis of the trabeculae at distal femur showed that the mineral apposition rate (MAR) and bone formation rate (BFR/BS) both declined with age, which were confirmed by the decreased width between xylenol and calcein labeling and reduced osteoid surface during aging in both models (Figs 1d and S1b–e, Supporting information). These data confirmed the presence of age‐related bone formation reduction in both models. In line with the above findings in human bones, we found an age‐related increase in PLEKHO1 protein expression and an age‐related decrease in the protein levels of total Smad1/5, p‐Smad1/5, and RUNX2 in bone tissues from both models (Fig. 1e,g). Consistently, the protein levels of RUNX2, the key osteogenic transcription factor that requires the BMP signaling for its induction of osteoblast phenotype (Phimphilai *et al*., 2006), were also decreased during aging. Moreover, the intraosseous protein levels of Smurf1 and Smurf2 remained unchanged during aging (Fig. 1e,g). In contrast, the levels of Smad1 ubiquitination at whole bone tissues were increased during aging (Fig. 1f,h). In addition, the *Plekho1* mRNA expression levels in either whole bone tissue or osteoblasts (ALP+ cells obtained at distal femora by laser‐captured microdissection) were upregulated in both models during aging (Figs 1i and S1f, Supporting information). Moreover, immunohistochemistry (IHC) analysis showed an age‐related increase in the instances of co‐localization of PLEKHO1+ with ALP+ cells at distal femora (Fig. 1j). Quantitatively, the ratio of PLEKHO1 and ALP co‐positive cells among the ALP+ cells at distal femora also increased with age in both models (Figs 1k and S1g, Supporting information). We further observed an age‐related decrease in the number of cells co‐expressing ALP and p‐Smad1/5 at the distal femur in the above rats (Figs 1l–m and S1h, Supporting information). Collectively, the data from human and rodents bone samples concordantly suggest that the aberrantly increased expression of PLEKHO1 within osteoblasts correlates with the decreased Smad‐dependent BMP signaling and reduced bone formation during aging.

### *Plekho1*‐depleted osteoblasts show enhanced osteogenic activity and promoted Smad‐dependent BMP signaling *in vitro*

**Figure 2:**
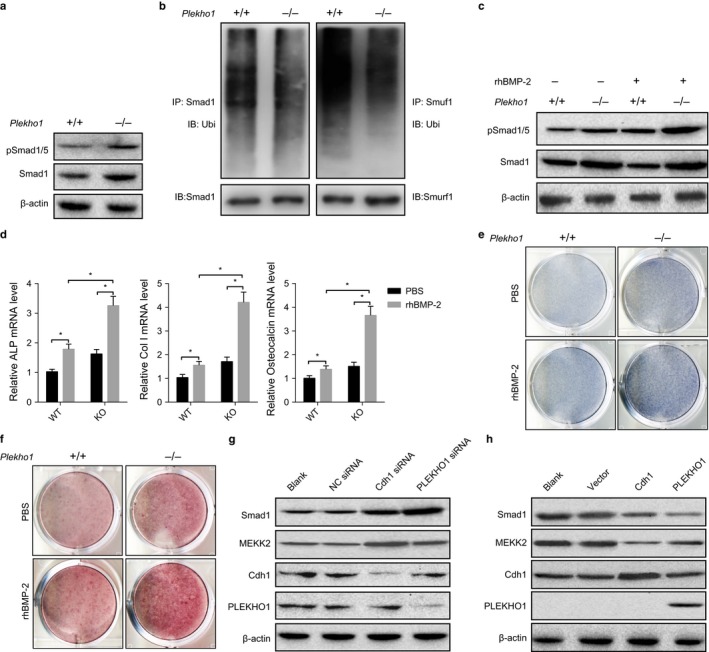
Enhanced Smad‐dependent BMP signaling and osteogenic differentiation in primary osteoblasts with PLEKHO1 deletion. (a) Levels of Smad1 and p‐Smad1/5 in primary osteoblasts isolated from WT or *Plekho1* knockout mice, as determined by immunoblot analysis. (b) Ubiquitylation of Smad1 and Smurf1 in primary osteoblasts isolated from WT or *Plekho1* knockout mice. Polyubiquitinated Smad1 and Smurf1 were detected by anti‐ubiquitin immunoblot analysis after precipitation of Smad1 and Smurf1 from osteoblasts pretreated with proteasome inhibitor MG132. (c) Levels of Smad1 and p‐Smad1/5 in primary osteoblasts isolated from WT or *Plekho1* knockout mice, in the absence or presence of rhBMP‐2. (d) Relative mRNA level of osteogenic differentiation markers (ALP, Col I, and osteogenic) in primary osteoblasts isolated from WT or *Plekho1* knockout mice, in the absence or presence of rhBMP‐2. (e) ALP staining showing ALP activity of primary osteoblasts isolated from WT or *Plekho1* knockout mice, in the absence or presence of rhBMP‐2. (f) Alizarin red staining showing mineralized nodule formation in primary osteoblasts isolated from WT or *Plekho1* knockout mice, in the absence or presence of rhBMP‐2. (g) Levels of Smad1 and MEKK2 in primary osteoblasts isolated from WT mice, in the absence of negative control siRNA (NC siRNA) or PLEKHO1 siRNA or Cdh1 siRNA. (h) Levels of Smad1 and MEKK2 in primary osteoblasts isolated from *Plekho1* knockout mice, in the absence of empty vector, or overexpression vector for PLEKHO1 or overexpression vector for Cdh1. *Note:* All data are the mean ± SD. *n *= 3 per group. *P < 0.05. Two‐way analysis of variance (ANOVA) with a Turkey's multiple comparisons test was performed to determine intergroup differences.

To explore the role of *Plekho1* in regulating the canonical Smad‐dependent BMP signaling in osteoblasts, we isolated the primary calvarial osteoblasts from neonates of the previously established *Plekho1* knockout (*Plekho1* ^−/−^) mice and *Plekho1 intact* (*Plekho1* ^*+*/*+*^) controls, respectively. As revealed by Western blot analysis, the *Plekho1* ^−/−^ osteoblasts had higher protein levels of Smad1 and pSmad1/5 as well as lower ubiquitination levels of Smad1 when compared to those in *Plekho1* ^*+*/*+*^ osteoblasts (Fig. 2a,b). Moreover, in response to rBMP‐2 stimulation, the *Plekho1* ^−/−^ osteoblasts showed more significant induction of Smad1 and pSmad1/5 and upregulated bone formation marker genes as well as higher levels of ALP activity and more mineralized nodule when compared to the *Plekho1* ^*+*/*+*^ osteoblasts (Fig. 2c–f). It is known that PLEKHO1 could also promote the Smurf1‐mediated ubiquitination of MEKK (Lu *et al*., 2008), a molecular that mediates the noncanonical BMP/JNK signaling (Yamashita *et al*., 2005). In addition, a recent study also reported that Cdh1 could also augment the Smurf1 activity and had an even more pronounced impact on the protein level of MEKK than PLEKHO1 *in vitro* (Wan *et al*., 2011). Therefore, we next sought to determine to which extent PLEKHO1 contributes to the protein levels of Smad1/5 and MEKK, respectively. Interestingly, in primary WT osteoblasts, we found that silencing PLEKHO1 could result in higher protein level of Smad1 than silencing Cdh1, whereas silencing Cdh1 could result in higher protein level of MEKK than silencing PLEKHO1 (Fig. 2g). In *Plekho1* ^−/−^ osteoblasts, we further documented that forced expression of *Plekho1* but not *Cdh1* could remarkably downregulate the protein level of Smad1, whereas forced expression of *Cdh1* also downregulates the protein level of MEKK (Fig. 2h). These findings suggest that PLEKHO1 could directly regulate the Smad‐dependent BMP signaling in osteoblasts. On the other hand, we found no significant differences in the protein levels of either phosphorylated BMP type Iα receptor (pBRIα), BRIα, or CK2 and the binding of CK2 and BRIα between the *Plekho1* ^−/−^ and *Plekho1* ^*+*/*+*^ osteoblasts (Fig. S2, Supporting information), suggesting that PLEKHO1 may not regulate the BMP signaling in osteoblasts in the CK2‐mediated manner.

### Osteoblast‐specific *Plekho1* depletion alleviates the age‐related reduction in Smad‐dependent BMP signaling and bone formation *in vivo*

To examine the effect of osteoblast‐targeted depletion of *Plekho1* gene on BMP signaling and bone formation during aging, we created the heterozygous mice carrying the mutant allele with *LoxP* sites harboring exon 3 to exon 6 of *Plekho1* gene (*Plekho1* ^*fl*/−^), which were then crossed with *Osx‐Cre* mice to generate the osteoblast‐specific *Plekho1* knockout mice (*Osx;Plekho1* ^*fl*/*fl*^, hereafter CKO mice) (Fig. S3a,b, Supporting information). As expected, the CKO mice presented remarkably lower *Plekho1* mRNA expression uniquely in bone tissue when compared to the *Plekho1* ^*fl*/*fl*^ mice (hereafter WT mice), whereas no significant differences in *Plekho1* mRNA expression were found in nonskeletal tissues between CKO and WT mice (Fig. S3c, Supporting information). Furthermore, the *Plekho1* mRNA expression was hardly detected in the ALP+ cells (osteoblasts) from CKO mice when compared to WT mice, whereas no significant differences in *Plekho1* mRNA expression within ALP‐ cells (nonosteoblasts) were found between CKO and WT mice (Fig. S3c, Supporting information).

**Figure 3:**
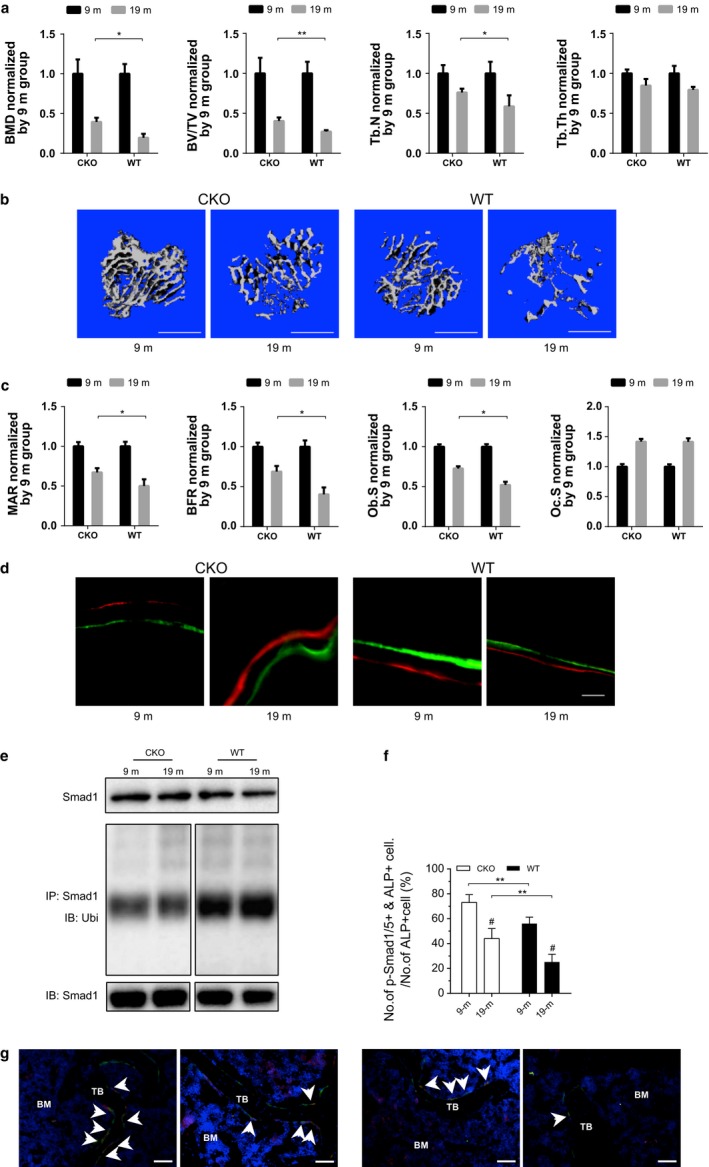
Attenuated decrease in bone formation in OVX osteoblast‐specific *Plekho1* knockout mice during aging. (a) The age‐related changes of micro‐CT parameters at proximal tibiae from *Osx;Plekho1* ^*fl*/*fl*^ (CKO) and *Plekho1* ^*fl*/*fl*^ (WT) mice during aging after OVX. (b) The representative 3D micro‐CT images of trabecular micro‐architecture at proximal tibiae from CKO and WT mice after OVX at the indicated time point. Scale bar = 1 mm. (c) The age‐related changes of bone histomorphometric parameters at proximal tibiae from CKO and WT mice during aging after OVX. (d) Representative micrographs of newly mineralized bone assessed by both xylenol (red) and calcein (green) labeling at proximal tibiae from CKO and WT mice after OVX at the indicated time point. Scale bar = 5 μm. (e) The representative electrophoretic bands of the total Smad1 protein level and ubiquitination level of Smad1 in 2–4 lumbar vertebras (LV 2–4) from the CKO and WT mice at 9 and 19 months of age, respectively. The mice were treated with MG132 (2 mg kg^−1^) through intraperitoneal injection 24 h before sample collections. (f) The age‐related changes of the ratio of pSmad1/5 and ALP co‐positive cells (pSmad1/5+ & ALP+) among the ALP+ cells at proximal tibiae from CKO and WT mice at 9 and 19 months of age, respectively. (g) The representative fluorescence micrographs of the protein expression of pSmad1/5 (red) in ALP+ (green) cells from CKO and WT mice at each time point. Scale bar = 100 μm. Arrow indicates the cells co‐expressing pSmad1/5 and ALP. *Note:* All data are the mean ± SD. *P < 0.05, **P < 0.01. #*P *< 0.05 vs. 9m. Two‐way analysis of variance (ANOVA) with a Turkey's multiple comparisons test was performed.

Next, the female CKO and WT mice were both ovariectomized at 4 months of age and sequentially sacrificed at 9 and 19 months of age, respectively. Micro‐CT analysis at the proximal tibiae showed that, although the BMD, BV/TV, trabecular number (Tb.N), and trabecular thickness (Tb.Th) were all decreased from 9 to 19 months of age after OVX, wherein the percentage of decrease in the former three parameters during aging was significantly lower in CKO mice as compared to WT mice (Fig. 3a and Table S2, Supporting information). Consistently, the CKO mice exhibited higher bone mass and more trabeculae as compared to WT mice at each time point, wherein the trabeculae structure was better preserved in CKO mice during aging after OVX (Fig. 3b). The bone histomorphometric analysis showed that the age‐related decreases in mineral apposition rate (MAR), bone formation rate (BFR/BS), and osteoblast surface (Ob.S/BS) were rapid in WT mice but mild in CKO mice after ovariectomy (Fig. 3c,d and Table S2, Supporting information). We also observed similar difference in the bone phenotypes between the female CKO and WT mice with sham operation during aging (Fig. S4a,b, Supporting information) as well as between the male CKO and WT mice during aging (Fig. S4c–f, Supporting information). The above findings indicated that PLEKHO1 within osteoblasts might inhibit bone formation and subsequently resulted in the impaired trabecular micro‐architecture and decreased bone mass during aging. On the other hand, we found that the intraosseous protein levels of total Smad1 were higher while the levels of ubiquitination Smad1 were lower in CKO mice when compared to WT mice at each time points (Fig. 3e). Moreover, the protein levels of total Smad1 were decreased while the ubiquitination levels of Smad1 were increased in bone specimens of WT mice from 9 to 19 months of age after OVX, whereas no obvious changes in either the total Smad1 protein levels or ubiquitination levels of Smad1 were found in CKO mice (Fig. 3e). In addition, IHC analysis showed the CKO mice had dramatically more cells co‐expressing p‐Smad1/5 and ALP at the proximal tibiae than those in WT mice at each time points (Fig. 3f,g). Collectively, it is suggested that targeted depletion of *Plekho1* gene in osteoblasts could attenuate the age‐related reduction in Smad‐dependent BMP signaling and bone formation.

### Osteoblast‐specific *Smad1* overexpression attenuates the age‐related bone formation reduction *in vivo*

**Figure 4:**
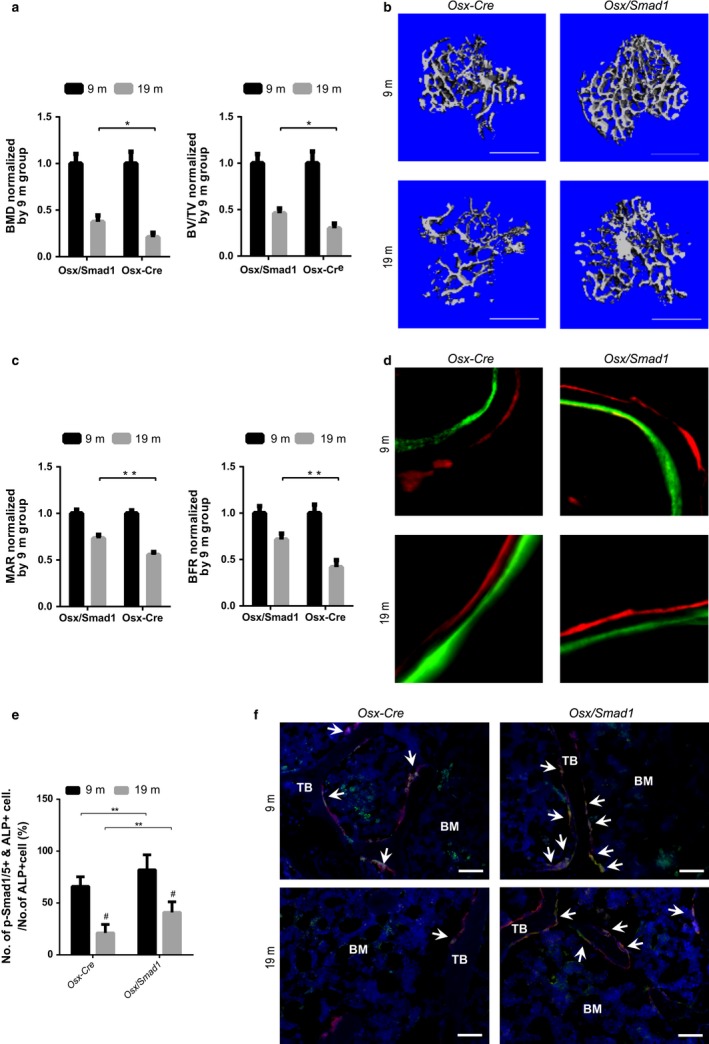
Attenuated bone formation reduction in osteoblast‐specific *Smad1* knock‐in (*Osx*/*Smad1*) mice during aging after ovariectomy. (a) The age‐related changes of micro‐CT parameters at proximal tibiae from *Osx*/*Smad1* mice and relative controls (*Osx‐Cre* mice) after ovariectomy (OVX), respectively. (b) The representative 3D micro‐CT images of trabecular micro‐architecture at proximal tibiae from *Osx*/*Smad1* mice and *Osx‐Cre* mice after OVX at the indicated time point, respectively. Scale bar = 1 mm (c) The age‐related changes of bone histomorphometric parameters at proximal tibiae from *Osx*/*Smad1* mice and *Osx‐Cre* mice after OVX, respectively. (d) The representative micrographs of newly mineralized bone assessed by both xylenol (red) and calcein (green) labeling at proximal tibiae from *Osx*/*Smad1* mice and *Osx‐Cre* mice after OVX at the indicated time point, respectively. Scale bar = 5 μm. (e) The age‐related changes of the ratio of pSmad1/5 and ALP co‐positive cells (PLEKHO1+ & ALP+) among the ALP+ cells at distal femur from *Osx*/*Smad1* mice and *Osx‐Cre* mice after OVX, respectively. (f) The representative fluorescence micrographs of the protein expression of pSmad1/5 (green) in ALP+ (red) cells at distal femora from *Osx*/*Smad1* mice and *Osx‐Cre* mice after OVX at the indicated time point, respectively. Scale bar = 50 μm. Arrow indicates the cells co‐expressing pSmad1/5 and ALP. BM: bone marrow; TB: trabecular bone. *Note:* *P < 0.05, **P < 0.01, ^#^ *P *< 0.05 vs. 9m group. One‐way analysis of variance (ANOVA) with a *post hoc* test was performed.

If the PLEKHO1‐mediated Smad1/5 ubiquitination and degradation could result in reduced bone formation during aging, then upregulation of Smad1 proteins in osteoblasts would probably have beneficial effect on bone formation. To test the above hypothesis, we created another mouse strain carrying the ROSA26‐PCAG‐STOP^flox^‐*Smad1*‐mCherry knock‐in allele, which were intercrossed with the *Osx‐Cre* mice to generate *Osx*/*Smad1* mice in which *Smad1* was specifically overexpressed in osteoblasts (Fig. S5a,b, Supporting information). IHC analysis of the cryosection at proximal tibiae showed that almost all the Smad1+ (mCherry+) cells were co‐localized with the ALP+ cells, indicating that the knock‐in exogenous *Smad1* gene was specifically expressed in ALP+ osteoblasts. In addition, the *Osx*/*Smad1* mice had remarkably higher *Smad1* mRNA expression in bone tissue but not in nonskeletal tissues when compared to that in control littermates (*Osx‐Cre*). We also detected notably higher *Smad1* mRNA expression in osteoblasts (mCherry+ cells) than that in nonosteoblasts (mCherry‐ cells) in *Osx*/*Smad1* mice (Fig. S5d, Supporting information). Next, we performed ovariectomy on both the *Osx*/*Smad1* mice and control littermates at 4 months of age and sequentially sacrificed them at 9 and 19 months of age, respectively. Micro‐CT analysis of the proximal tibiae showed that the percentage of decrease in BMD and BV/TV was both significantly lower in *Osx*/*Smad1* mice than those in their control littermates during aging (Fig. 4a and Table S2, Supporting information). Consistently, the *Osx*/*Smad1* mice showed improved trabecular structure and higher bone mass than their littermates at each time point (Fig. 4b). In addition, bone histomorphometric analysis of the proximal tibiae revealed that the percentage of decrease in MAR and BFR/BS in *Osx*/*Smad1* mice was also significantly lower than those in their littermates (Fig. 4c,d and Table S2, Supporting information). Furthermore, IHC analysis revealed more instances of co‐localization of pSmad1/5+ with ALP+ cells at proximal tibiae from *Osx*/*Smad1* mice when compared to those from control littermates at each time point (Fig. 4e,f). Collectively, these results suggest that overexpression of *Smad1* could attenuate the age‐related reduction in bone formation.

### Overexpression of *Plekho1* in osteoblasts counteracts the beneficial effect of osteoblast‐specific *Smad1* overexpression on bone formation during aging

**Figure 5:**
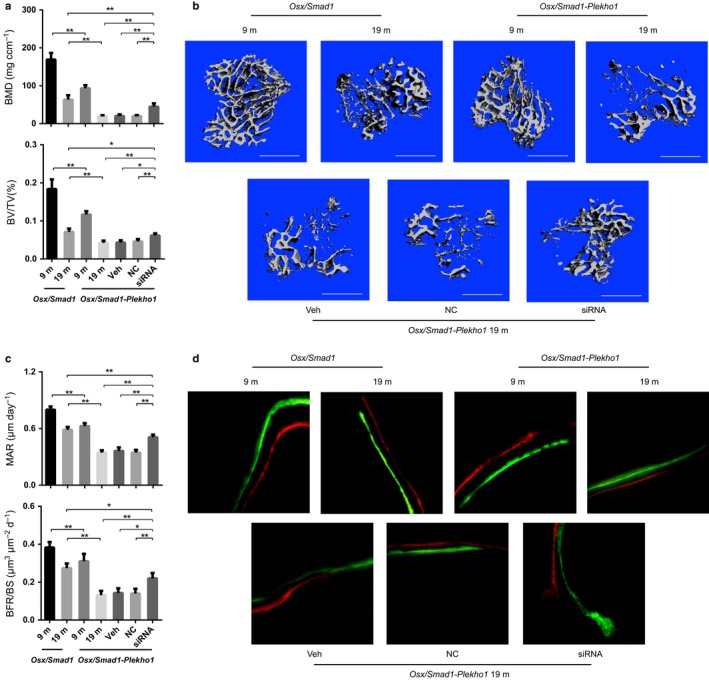
Progressive bone formation reduction in osteoblast‐specific *Smad1 and Plekho1* double knock‐in (*Osx*/*Smad1‐Plekho1*) mice during aging after ovariectomy and partial rescue by osteoblast‐targeted *Plekho1* siRNA treatment. (a) The age‐related changes of micro‐CT parameters at proximal tibiae from *Osx*/*Smad1*,* Osx*/*Smad1‐Plekho1*, vehicle (Veh), nonsense RNA control (NC), and siRNA groups after ovariectomy (OVX), respectively. (b) Representative 3D micro‐CT images of trabecular micro‐architecture at proximal tibiae from *Osx*/*Smad1*,* Osx*/*Smad1‐Plekho1*, vehicle (Veh), NC, and siRNA groups after OVX at the indicated time point, respectively. Scale bar = 1 mm (c) Age‐related changes of bone histomorphometric parameters at proximal tibiae from *Osx*/*Smad1*,* Osx*/*Smad1‐Plekho1*, vehicle (Veh), NC, and siRNA groups after OVX, respectively. (d) Representative micrographs of newly mineralized bone assessed by both xylenol (red) and calcein (green) labeling at proximal tibiae from *Osx*/*Smad1*,* Osx*/*Smad1‐Plekho1*, vehicle (Veh), NC, and siRNA groups after OVX at the indicated time point, respectively. Scale bar = 5 μm. *Note:* All data are the mean ± SD. *P < 0.05, **P < 0.01. One‐way analysis of variance (ANOVA) with a *post hoc* test was performed.

To investigate whether PLEKHO1 could promote ubiquitination and degradation of Smad1/5 to dampen the BMP signaling to inhibit bone formation, we created another mouse strain carrying the ROSA26‐PCAG‐STOP^fl^‐*Plekho1*‐eGFP allele (Fig. S6a,b, Supporting information), and crossed them with the aforementioned *Osx*/*Smad1* mice to generate the *Osx*/*Smad1‐Plekho1* mice that co‐overexpressing *Smad1* and *Plekho1* in osteoblasts. IHC analysis of the cryosection at proximal tibiae from *Osx*/*Smad1‐Plekho1* mice showed that the Smad1+ (mCherry+) cells were co‐localized with the PLEKHO1+ (eGFP+) cells (Fig. S5c, Supporting information), suggesting that the knock‐in exogenous *Smad1* and *Plekho1* genes were simultaneously expressed in osteoblasts. Besides, the *Osx*/*Smad1‐Plekho1* mice had remarkably higher *Smad1* and *Plekho1* mRNA levels in bone tissue and mCherry and eGFP co‐positive cells than those in nonskeletal tissues from control littermates and mCherry/eGFP negative cells from *Osx*/*Smad1‐Plekho1* mice, respectively (Fig. S5d,e, Supporting information). Thereafter, the *Osx*/*Smad1* and *Osx*/*Smad1‐Plekho1* mice were both ovariectomized at 4 months of age and sequentially sacrificed at 9 and 19 months of age, respectively. Micro‐CT analysis of the proximal tibiae showed that the values of BMD and BV/TV were both significantly lower in *Osx*/*Smad1‐Plekho1* mice than those in *Osx*/*Smad1* mice at 9 and 19 months of age, respectively (Fig. 5a). Consistently, the *Osx*/*Smad1‐Plekho1* mice showed poorer trabecular structure and lower bone mass than *Osx*/*Smad1* mice at each time point (Fig. 5b). In addition, bone histomorphometric analysis of the proximal tibiae revealed that the MAR and BFR/BS in *Osx*/*Smad1‐Plekho1* mice were both significantly lower than those in *Osx*/*Smad1* mice at each time points (Fig. 5c,d).

To determine whether the upregulated PLEKHO1 expression was responsible for the age‐related bone phenotype in *Osx*/*Smad1‐Plekho1* mice, we next evaluated the effect of osteoblast‐targeted *Plekho1* siRNA treatment on bone formation in *Osx*/*Smad1‐Plekho1* mice during aging. Another batch of *Osx*/*Smad1‐Plekho1* mice was ovariectomized at 4 months of age and left untreated until 9 months of age. Then, the mice were treated with either *Plekho1* siRNA encapsulated in our previously established osteoblast‐targeted delivery system (Zhang *et al*., 2001), that is, *Plekho1* siRNA‐(AspSerSer)_6_‐liposome (siRNA group), nonsense siRNA‐DSS_6_‐liposome (NC group), or (AspSerSer)_6_‐liposome alone (Veh group) at an interval of 2 weeks and sacrificed at 19 months of age. The *Osx*/*Smad1‐Plekho1* mice from siRNA group had significantly higher values of micro‐CT and histomorphometry parameters at the tibiae metaphysis when compared to those in the mice from NC and Veh groups (Fig. 5a–d). Collectively, the above results suggest that upregulation of *Plekho1* in osteoblasts could counteract the beneficial effect of osteoblast‐specific *Smad1* overexpression on bone formation during aging.

### Enhanced Smad‐dependent BMP signaling, promoted bone formation, and increased bone mass after therapeutic silencing PLEKHO1 within osteoblasts in aging rats

**Figure 6:**
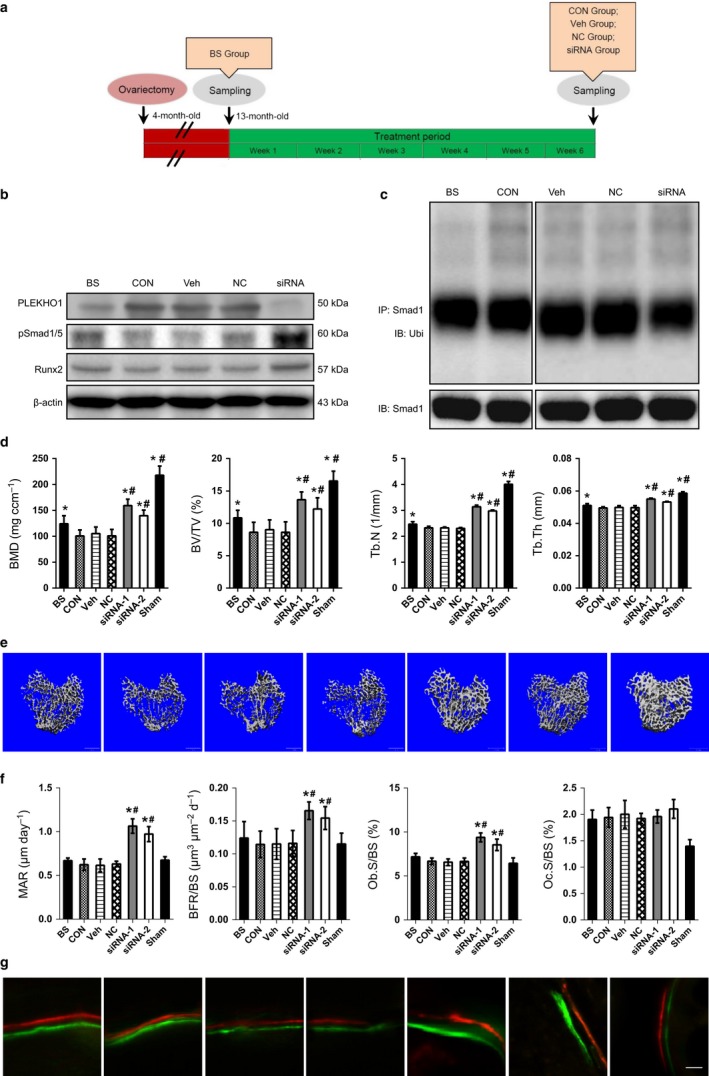
Enhanced Smad‐dependent BMP signaling, promoted bone formation, and increased bone mass by osteoblast‐targeting PLEKHO1 siRNA treatment in aging ovariectomized rats. (a) A schematic diagram illustrating the experimental design. BS: Baseline group sacrificed before treatment initiation. CON: Control group treated with PBS alone. Veh: Vehicle control group treated with (AspSerSer)_6_‐liposome alone. NC: nonsense siRNA control group treated with (AspSerSer)_6_‐liposome‐NC siRNA. siRNA: (AspSerSer)_6_‐liposome‐PLEKHO1 siRNA treated group. (b) Western blot analysis of PLEKHO1, pSmad1/5, and RUNX2 in the indicated group. Upper panel: The representative electrophoretic bands. Lower panel: The semiquantitative data of the protein levels. (c) The representative electrophoretic bands for the ubiquitination levels of Smad1 in 2nd ~ 4th lumbar vertebrates (LV 2–4) from the rats in the indicated group. The rats were treated with MG132 (2 mg kg^−1^) through intraperitoneal injection 24 h before sample collections. (d) The quantitative data of micro‐CT parameters at proximal tibiae in the indicated group. (e) The representative 3D micro‐CT images of trabecular architecture at proximal tibiae in the indicated group. Scale bar = 1 mm (f) The quantitative data of bone histomorphometric parameters at proximal tibiae in the indicated group. (g) The representative micrographs of newly mineralized bone assessed by both xylenol (red) and calcein (green) labeling at proximal tibiae in each group. Scale bar = 10 μm. *Note:* All data are the mean ± SD. *P < 0.05 vs. either CON, NC, or Veh group. #*P *< 0.05 vs. BS group. One‐way analysis of variance (ANOVA) with a *post hoc* test was performed.

Thereafter, we investigated the effect of therapeutic silencing *Plekho1* within osteoblasts on BMP signaling and bone formation in two aging rodent models (the OVX rats and male rats). Two independent PLEKHO1 siRNA sequences that have been validated in our previously published studies (Zhang *et al*., 2001; Guo *et al*., 2008) were encapsulated into the osteoblast‐targeting delivery system, *that is,* (AspSerSer)_6_‐liposome. Then, we performed periodic systemic administration of PLEKHO1 siRNA (3.75 mg kg^−1^) through tail vein injection for 6 weeks either in OVX rats at 9 months after ovariectomy (13 months of age) or in male rats at 16 months of age (Figs 6a and S7a, Supporting information). We found that the intraosseous PLEKHO1 protein expression levels in siRNA group were remarkably lower, whereas the intraosseous pSmad1/5 and RUNX2 protein levels in siRNA group were higher than those in CON, NS, or Veh group (Fig. 6b). Accordingly, the levels of Smad1 ubiquitination were significantly lower in siRNA group when compared to those in CON, NS, or Veh group (Fig. 6c). The micro‐CT analysis of the proximal tibiae showed that the BMD, BV/TV Tb.N, and Tb.Th in two siRNA groups were all significantly higher than baseline but lower than those in sham‐operated controls. In contrast, the above three parameters in CON, NC, and Veh groups were all significantly lower than baseline (Figs 6d and S7b, Supporting information). Consistently, a better organized trabecular micro‐architecture and a higher bone mass at proximal tibiae were also observed in the two siRNA groups as compared to the rats in BS, CON, NC, and Veh groups (Figs 6e and S7c, Supporting information). The bone histomorphometric data showed that the MAR, BFR, and Ob.S/BS in the two siRNA groups were all significantly increased from baseline and higher than those in NC, Veh, and CON groups as well as sham groups (Figs 6f and S7d, Supporting information). We also found extensive xylenol and calcein labeling and a larger width between the two labeling bands in the two siRNA groups when compared to those in BS, NC, Veh, and CON groups (Figs 6g and S7e, Supporting information). It is suggested that therapeutic silencing *Plekho1* within osteoblasts could enhance the Smad‐dependent BMP signaling, promote bone formation, and increase bone mass in aging rats.

## Discussion

The present study showed that the aberrantly high PLEKHO1 expression in osteoblasts is responsible for the age‐related bone formation reduction. By genetic approach, we demonstrated that PLEKHO1 could promote the ubiquitination of Smad1 to suppress the Smad‐dependent BMP signaling in osteoblasts and inhibit bone formation during aging. Furthermore, we showed that therapeutic silencing PLEKHO1 in osteoblast could significantly enhance Smad‐dependent BMP signaling, promote bone formation, and increase bone mass in aging rodents.

### Increased PLEKHO1 expression in osteoblasts contributes to the reduction in Smad‐dependent BMP signaling and bone formation during aging

In this study, we found an age‐related increase in the expression of PLEKHO1 either in bone specimens from fractured elders or in both bone specimens and osteoblasts from aging rats (both OVX rats and male rats). In addition, the elevated PLEKHO1 expression was accompanied by the reduced intraosseous Smad‐dependent BMP signaling, as evidenced by the increased ubiquitination of Smad1 and decreased protein levels of total Smad1/5 and pSmad1/5 either in bone specimens from fractured elders and aging rats. More importantly, the elevated PLEKHO1 expression was closely associated with reduced bone formation during aging, as evidenced by either the age‐related reduction in the expression of bone formation marker gene (ALP) in bone specimens from the fractured elders or the age‐related decrease in bone formation‐related histomorphometric parameters (MAR and BFR/BS) from the aging rats. Furthermore, we found that osteoblast‐specific *Plekho1* gene depletion could notably alleviate the age‐related decrease in pSmad1/5 protein levels and increase in Smad1 ubiquitination, as well as attenuate the age‐related decrease in both micro‐CT and bone histomorphometry parameters in CKO mice when compared to their WT controls. All these results indicate that the aberrantly elevated PLEKHO1 expression in osteoblasts contributes to the reduction in Smad‐dependent BMP signaling and bone formation during aging.

### Reduced Smad‐dependent BMP signaling contributes to bone formation reduction during aging

It is well accepted that the normal Smad‐dependent BMP signaling is indispensable for the postnatal bone development (Retting *et al*., 2009; Song *et al*., 2009; Wang *et al*., 2011; Chen *et al*., 2012). However, there is no report on the impact of aging on the Smad‐dependent BMP signaling in bone. In the current study, we provided the first evidence from aging human bone samples to show that the protein levels pSmad1/5 were decreased with age. We further observed the age‐related decrease in the Smad‐dependent BMP signaling within osteoblasts in aging rats. Additionally, we employed the osteoblast‐specific *Smad1* overexpression (*Osx*/*Smad1*) mice to determine the impact of Smad1 gain of function on bone formation during aging. We found higher bone mass and improved microarchitectures in the OVX *Osx*/*Smad1* mice at either 9 or 19 months of age when compared to those in the control littermates. Moreover, the age‐related decrease in the values of micro‐CT and bone histomorphometry parameters was remarkably attenuated in the OVX *Osx*/*Smad1* mice. It has been previously reported that the osteoblast‐specific *Smad1* knockout mice had partially dampened BMP signaling and developed an osteopenic phenotype (Wang *et al*., 2011). Collectively, the above findings from both gain‐ and loss‐of‐function genetic models suggest that the age‐related downregulation of Smad‐dependent BMP signaling could lead to the reduced bone formation during aging.

### Increased PLEKHO1 expression in osteoblasts promotes the ubiquitination of Smad1 to suppress the Smad‐dependent BMP signaling and inhibit bone formation during aging

Smurf1 is a well‐recognized molecule to negatively regulate bone formation *via* mediating the ubiquitination of BMP signaling proteins (Zhao *et al*., 2004; Yamashita *et al*., 2005; Guo *et al*., 2014; Xing *et al*., 2010). It was also demonstrated that Smurf1 could promote the proteasomal degradation of BMP signaling proteins to mediate the TNF‐induced systemic bone loss (Guo *et al*., 2014). Smurf2 is also previously recognized as a negative regulator of Smad1 (Zhang *et al*., 2013). However, unlike PLEKHO1, we found no significant change in the expression of either Smurf1 or Smurf2 in the aging bone, which is consistent with a previous study showing no obvious difference in the mRNA expression of Smurf1 in bone marrow‐derived osteoprogenitors and bone samples from mice during aging (Xing *et al*., 2010; Shu *et al*., 2013). Therefore, it suggests that Smurf1 and Smurf2 may not contribute as much to the age‐related reduction in Smad‐dependent BMP signaling and bone formation as PLEKHO1. In addition, although *Plekho1* ^−/−^ osteoblasts showed promoted MEKK‐mediated BMP/JNK signaling *in vitro* (Lu *et al*., 2008), the change of Smad‐dependent BMP signaling in *Plekho1* knockout osteoblasts was undetermined. On the other hand, a recent study found that depletion of endogenous *Cdh1*, another ubiquitination‐related molecule that could also augment Smurf1 activity (Wan *et al*., 2011; Kannan *et al*., 2012), could cause a more dramatic induction of MEKK2 than depletion of *Plekho1* in HeLa cells (Wan *et al*., 2011). It further showed that ectopic expression of *Cdh1*, but not *Plekho1*, could suppress MEKK2 induction in Cdh1‐depleted HeLa cells, whereas ectopic expression of Cdh1 also suppresses MEKK2 induction in PLEKHO1‐depleted HeLa cells (Wan *et al*., 2011), implying that PLEKHO1‐dependent regulation may not be the dominant regulatory mechanism for the MEKK2‐mediated non‐canonical BMP signaling. In the *in vitro* study, we further observed promoted Smad‐dependent BMP signaling in the *Plekho1* knockout osteoblasts. Intriguingly, silencing PLEKHO1 had more pronounced effect on induction of Smad1 than silencing Cdh1 in primary osteoblasts. Moreover, we found that forced expression of *Plekho1* but not *Cdh1* could dramatically suppress the Smad‐dependent BMP signaling induction in *Plekho1* ^−/−^ osteoblasts. These findings indicate that PLEKHO1 could directly regulate the Smad‐dependent BMP signaling in osteoblasts. In addition, it has been shown that PLEKHO1 is responsible for recruitment of CK2 to the plasma membrane (Olsten *et al*., 2004), where CK2 could interact with BRIa to negatively regulate BMP signaling (Bragdon *et al*., 2010). However, we found no significant differences in the protein expression of either BRIa or CK2 between the *Plekho1* ^−/−^ and *Plekho1* ^*+*/*+*^ osteoblasts, suggesting that PLEKHO1 may not regulate the BMP signaling in a CK2‐mediated manner within osteoblasts. Thereafter, we genetically increased the expression of *Plekho1* within osteoblasts on the background of *Osx*/*Smad1* mice. Impressively, when compared to the OVX *Osx*/*Smad1* mice, the OVX *Osx*/*Smad1‐Plekho1* mice showed accelerated reduction in bone formation during aging. More importantly, the above harmful effect caused by *Plekho1* overexpression could be partially rescued after knocking down the intraosteoblast PLEKHO1 level by osteoblast‐targeted PLEKHO1 siRNA treatment. Taken all together, it suggests that the elevated PLEKHO1 in osteoblasts could promote the ubiquitination of Smad1 to suppress the Smad‐dependent BMP signaling and inhibit bone formation during aging.

### Therapeutic silencing PLEKHO1 in osteoblasts may be a potential bone anabolic strategy to reverse established osteoporosis during aging

To date, intermittent injection of recombinant human parathyroid hormone (iPTH) is the only FDA‐approved anabolic agents for stimulating bone formation (Neer *et al*., 2001; Marie & Kassem, 2011). However, concerns over the increased bone resorption after 2‐year period of treatment limited the further clinical application of iPTH in treating age‐related bone formation reduction (Black & Schafer, 2013). Thus, it is highly desirable to search for new and safe bone anabolic agent that does not activate bone resorption. Our results demonstrated that silencing *Plekho1* within osteoblast by administration of *Plekho1* siRNA encapsulated within the osteoblast‐specific delivery system could significantly enhance the Smad‐dependent BMP signaling, promote bone formation, and increase bone mass in both aging OVX rats and aging male rats. In addition, we found that manipulating *Plekho1* within osteoblast by either genetic or pharmacological approaches had minimum effect on bone resorption. Collectively, it indicates that silencing PLEKHO1 in osteoblasts would exert anabolic effect to promote bone formation without potential induction on bone resorption. Therefore, the intraosteoblast PLEKHO1 would be an ideal therapeutic target for treating the age‐related bone formation reduction.

In conclusion, our study established the critical role of intraosteoblast PLEKHO1 in the pathophysiological mechanism of age‐related bone formation reduction. Toward translational medicine, it indicates that targeting PLEKHO1 in osteoblasts may be a potential bone anabolic strategy to reverse established osteoporosis during aging.

## Evaluation protocols

### Human bone specimen preparation

We collected femoral head cancellous bone specimens from 40 aged patients with hip fractures at the age between 60 and 90 years old. Patients who had fracture caused by falling without obvious violence were included in our study (inclusive criteria). Subjects with malignancy, diabetes, or other severe diseases in the previous 5 years were excluded from our study (exclusive criteria). All the clinical procedures were approved by the Ethics Committees of the university. We also obtained informed consent from the participants. The bone specimens were stored in liquid nitrogen until used for RNA and protein extractions.

### Animal model

To generate the *Plekho1* ^*fl*/*fl*^ mice and *Osx;Plekho1* ^*fl*/*fl*^ mice, firstly, a floxed allele of *Plekho1* was generated containing *LoxP* sites flanking exon 3–6 of *Plekho1*. Neo cassette flanked by *frt* sites was inserted downstream of exon 6. A 6.1 kb 5′ homologous arm and a 4.5 kb 3′ homologous arm were used for homologous recombination (Fig. S3a, Supporting information). The targeting vector is constructed, fully sequenced, and electroporated into embryonic stem (ES) cells. Positive targeting clones were identified by polymerase chain reaction (PCR) and Southern blotting. The targeted ES clones were microinjected into C57BL/6 blastocysts, and male chimeras were mated to C57BL/6 females to obtain *Plekho1* ^*fl‐neo*/*+*^ mice. *Plekho1* ^*fl‐neo*/*+*^ mice were then intercrossed with *flp* deleter mice (JAX, 009086) to remove neo cassette for generating *Plekho1* ^*fl*/*+*^ mice. Thereafter, we intercrossed *Plekho1* ^*fl*/*+*^ mice with *Osx‐Cre* mice (Beijing Biocytogen Co., Ltd, China, http://www.biocytogen.com/userfiles/ue/file/20160308/1457424897233028370.pdf) to generate *Osx;Plekho1* ^*fl*/*fl*^ and *Plekho1* ^*fl*/*fl*^ mice. To generate the *Osx*/*Smad1* mice and *Osx*/*Plekho1‐Smad1* mice, firstly, we generated the *Rosa26‐PCAG‐STOP* ^*fl*^‐*Smad1* knock‐in *R26‐Smad1*) mice. In brief, a cassette containing the following components was constructed to target the *Rosa26* locus: *FRT‐LoxP‐stop* codons‐three SV40 *poly(A)* sequences‐*LoxP‐Smad1‐mCherry‐WPRE‐bGH poly(A)‐AttB‐PGK* promoter‐*FRT‐Neo‐PGK poly(A)‐AttP* (Fig. S5a, Supporting information). The targeting vector is constructed, fully sequenced, and electroporated into embryonic stem (ES) cells. Positive targeting clones were identified by polymerase chain reaction (PCR) and Southern blotting. The targeted ES clones were microinjected into C57BL/6J blastocysts to obtain chimeric mice following standard procedures, which were then backcrossed to C57BL/6 background. Similarly, we also generated the *Rosa26‐PCAG‐STOP* ^*fl*^‐*Plekho1* knock‐in (*R26‐Plekho1*) mice (Fig. S6a, Supporting information). Thereafter, we crossed the *R26‐Smad1* mice with *Osx‐Cre* mice *and R26‐Plekho1* mice to obtain *Osx*/*Smad1* mice and *Osx*/*Plekho1‐Smad1* mice, respectively.

The Sprague Dawley rats and genetically modified mice were all maintained under standard animal housing conditions (12‐h light, 12‐h dark cycles and free access to food and water). The female rats and mice were ovariectomized or sham‐operated at 4 months of age, respectively. At the corresponding time points in each study, the OVX rats/mice were euthanized for sample collection. All the experimental procedures were approved by the Committees of Animal Ethics and Experimental Safety of Hong Kong Baptist University and the Chinese University of Hong Kong.

### Preparation of the osteoblast‐targeting delivery system encapsulating PLEKHO1 siRNA

The PLEKHO1 siRNA encapsulated in the osteoblast‐targeting delivery system, that is, (AspSerSer)_6_‐liposome‐siRNA, was prepared using our previously reported methods (Wan *et al*., 2011; Zhang *et al*., 2001). In brief, the liposomes were firstly prepared by lipid film method described as previous study (Kheirolomoom & Ferrara, 2007). Then, the (aspartate–serine–serine)_6_, that is, (AspSerSer)_6_, with an N‐terminal acetylcysteine residue was incubated with the preformed liposome for 2 h at ambient temperature with the molar ratio of (AspSerSer)_6_ to DSPE‐PEG2000‐MAL at 3:1. Subsequently, the liposome suspension was purified by size exclusion chromatography to remove the unconjugated (AspSerSer)_6_. Thereafter, equal volume of liposome suspensions in aliquots was mixed with distilled water containing mannitol and lyophilized for 48 h using freeze‐dryer (Labconco, Freezezone, Kansas City, MO, USA). Finally, the above lyophilized liposomes with 15 mmol lipids were rehydrated by adding another volume of DEPC‐treated water containing PLEKHO1 siRNAs (Shanghai GenePharma Co., Ltd, Shanghai, China) and were incubated for 20 min at room temperature. The entrapment procedure was performed immediately before use followed by sterilization through a 0.22‐μm sterile filter.

### Total RNA extraction, reverse transcription, and quantitative real‐time PCR

RNeasy Mini kit (Qiagen, Hong Kong, Cat. No. 74106) was used to extract total RNA from cultured cells and tissues using the commercialized protocol. For cultured cells, after aspirating the medium, the cells were washed with PBS and disrupted by adding 350 μL buffer RLT and homogenized by pipetting up and down. For tissues, 600 μL of buffer RLT was added directly to the tissues after crashed by pestle grinder. After centrifugation at 12,000 × g for 5 min, the supernatant was collected. Then, one volume of 70% ethanol was added to the homogenized lysate, mixed well, and transferred to an RNeasy spin column and spun for 15 s at 12,000 × *g* (10 000 rpm). After discarded the flow‐through, the spin column membrane was washed by adding 700 μL buffer RW1 and centrifuged for 15 s at 12,000 × *g* (10 000 rpm). Thereafter, the spin column membrane was washed twice by 500 μL buffer RPE and centrifuged for 15 s at 12,000 × *g* (10 000 rpm). Then, 50 μL RNase‐free water was added directly to the spin column membrane. After centrifuged for 1 min at 12,000 × *g* (10 000 rpm), the total RNA was eluted from the column. The yield and purity of RNA were measured by spectrophotometry.

Total RNA was reverse‐transcribed to cDNA using the following established protocol. A total volume of 12 μL reaction volume, including 1 μg total RNA, 1 μL 50 ng μL^−1^ random primers, and 1 μL dNTP Mix (10 mm each), was heated to 65 °C for 5 min and quick chill on ice. And then, the contents of the tube were collected by brief centrifugation and mixed with 4 μL 5× first‐strand buffer, 2 μL 0.1 m DTT and 1 μL RNaseOUT™ (40 units μL^−1^). Thereafter, the contents in the tube were mixed with 1 μL (200 units) of SuperScript™ II RT by pipetting gently up and down. After that, the mixture were incubated at 42 °C for 50 min to get the cDNA followed by inactivation at 70 °C for 15 min.

The 10 μL volume of the final quantitative real‐time PCR solution contained 1 μL of the diluted cDNA product, 5 μL of 2× Power SYBR^®^ Green PCR Master Mix (Applied Biosystems, Foster City, CA, USA), 0.5 μL each of forward and reverse primers, and 3 μL nuclease‐free water. The primer sequences are listed in Table S1 (Supporting information). The amplification conditions were as follows: 50 °C for 2 min, 95 °C for 10 min, 40 cycles of 95 °C for 15 s, and 60 °C for 1 min. The fluorescence signal emitted was collected by ABI PRISM^®^ 7900HT Sequence Detection System and the signal was converted into numerical values by sds 2.1 software (Applied Biosystems). The mRNA expression level of the target gene was first calculated from the relative standard curve method by the sds 2.1 software. The threshold cycle (CT) value, which represents the relative expression of each target gene, was determined from the corresponding curve. Then, the relative expression of mRNA was determined by dividing the target amount by endogenous control amount to obtain a normalized target value (Wang *et al*., 2013).

### Immunoprecipitation

For immunoprecipitation (IP), the bone tissues were homogenized on ice in IP lysis buffer and incubated on an orbital rotator at 4 °C for 24 h. Then, the homogenates were centrifugated at 12 000 *g* for 10 min. After total protein quantification, the cell lysates were subjected to magnetic IP (Pierce Classic Magnetic IP/Co‐IP Kit; Thermo Scientific, Hong Kong) according to the manufacturer's recommendation. Briefly, the cell lysates with a total of 500 μg protein were combined with 4 μg of anti‐Smad1 antibodies (Abcam, Hong Kong, ab131371), respectively, and incubated on an orbital rotator overnight at 4 °C to form the immune complex. Thereafter, the above antigen sample/antibody mixtures were added into a 1.5‐mL microcentrifuge tube containing the prewashed magnetic beads and incubated at room temperature for 1 h with mixing. Then, the beads were collected with a magnetic stand, while the supernatant with unbound antigens was saved as the input for the downstream Western blotting analysis. After that, the beads were washed twice with the IP wash buffer followed by another wash with ultrapure water. Then, the beads were mixed with the Western blot sample buffer and heated at 99 °C in a heating block for 10 min for elution of the combined target antigen (Smad1). Finally, the supernatant containing target antigen was magnetically separated from the beads and subjected to Western blotting analysis (Morel *et al*., 2013).

### Western blot analysis

Total proteins (40 μg) were loaded from each sample on denaturing SDS‐PAGE. Immunodetection of rat, human, and mouse PLEKHO1, total Smad1 and phosphorylated Smad1/5, as well as rat Smurf1 were done using anti‐PLEKHO1 antibody (1: 500; Santa Cruz Biotechnology, Santa Cruz, CA, USA), anti‐Smad1 antibody (1:1000; Abcam), anti‐Smad5 antibody (1:1000; Abcam, Hong Kong), anti‐RUNX2 antibody (1:1000; Abcam, Hong Kong), antiphosphorylated Smad1/5 antibody (1:1000; Cell Signaling Technology, Inc., USA), anti‐Smurf1 (1:500, Santa Cruz Biotechnology, CA, USA), and anti‐Smurf2 (1:500, Santa Cruz Biotechnology, CA, USA), respectively. Enhanced chemiluminescence was used for detection. To check the amount of proteins transferred to nitrocellulose membrane, β‐actin or GAPDH was used as control and detected by an anti‐β‐actin or anti‐GAPDH monoclonal antibody (1: 500; Sigma, USA). The relative amounts of the transferred proteins were quantified by scanning the autoradiographic films with a gel densitometer (Bio‐Rad, Hong Kong) and normalized to the corresponding β‐actin level. For immunodetection of the ubiquitination level of endogenous Smad1, the PVDF membrane was primarily probed with the anti‐ubiquitin antibody (1:1000; Abcam, Hong Kong) and secondarily incubated with the mouse anti‐rabbit IgG (conformation specific) antibody (1:1000; Cell Signaling Technology, Inc., USA) to eliminate the heavy and light chains on the blot.

### Laser‐captured Microdissection (LCM)

The right femora were decalcified in 10% EDTA and embedded in OCT. Then, the series frozen sections (5 μm) from proximal tibiae were prepared in a cryostat (CM3050; Leica Microsystems, Wetzlar, Germany) at −24 °C. The adjacent sections were mounted on either glass slides or polyethylene membrane‐equipped slides (P.A.L.M., Bernried, Germany). The sections mounted on glass slides were performed immunostaining to identify the ALP‐positive cells. Briefly, the cryosections were incubated overnight at 4 °C with rabbit polyclonal anti‐ALP antibody (1:50 dilution; Santa Cruz Biotechnology) after fixation and blocking. Then, the sections were incubated with Alexa Fluor 488‐conjugated donkey anti‐rabbit IgG (1:400 dilutions; Invitrogen, Hong Kong). Finally, the sections were mounted with medium containing DAPI (Vector Laboratories, Burlingame, CA, USA) and examined under a fluorescence microscope to identify the ALP‐positive staining cells. The adjacent sections mounted on membrane‐coated slides were stained with neutral red for 1 min at room temperature. After brief rinsing in water, the sections were air‐dried. ALP‐positive staining cells in adjacent sections were isolated by microdissection with an upgraded laser pressure catapulting microdissection system (P.A.L.M.) using a pulsed 355 nm diode laser in the Leica LMD 7000 Laser Microdissection System. About 100–200 identified cells were collected in reaction tube containing 5 μL lysis buffer for total RNA extraction and subsequent real‐time PCR analysis (Wang *et al*., 2013; Liang *et al*., 2015).

### Micro‐CT analysis

The tibiae were *ex vivo* scanned by micro‐CT system (*viva* CT40; Scanco Medical, Brüttisellen, Switzerland). A total of 426 slices at the proximal tibiae with a voxel size of 10 μm were scanned above/below the growth plate of the distal femora/proximal tibiae. The whole trabecular bone was isolated for three‐dimensional reconstruction (Sigma = 1.2, Supports = 2 and Threshold = 190) to calculate the following parameters: bone mineral density (BMD), relative bone volume (BV/TV), trabecular number (Tb.N), and trabecular thickness (Tb.Th) (Zhang *et al*., 2001).

### Bone histomorphometric analysis

The rats and mice were injected intraperitoneally with calcein green (10 mg kg^−1^) and xylenol orange (90 mg kg^−1^) at 10 and 2 days, respectively, before euthanasia. After sacrificing, the femurs or tibias were dehydrated in graded concentrations of ethanol and embedded without decalcification in the modified methyl methacrylate (MMA) using our previously established protocol (Qin *et al*., 2001). Briefly, the proximal tibiae were dehydrated in 70%, 85%, 90%, and 100% graded ethanol and then submerged in xylene for three times. Thereafter, the femora was submerged in MMA‐I (60% methyl methacrylate, 35% butyl methacrylate, 3.8% methyl benzoate, and 1.2% polyethylene glycol), MMA‐II (0.4 g benzoyl peroxide added per 100 mL MMA‐I), and MMA‐III (0.8 g benzoyl peroxide added per 100 mL MMA‐I) in turn. Finally, the proximal tibiae were solidified in the MMA‐III added with accelerator of N, N‐dimethyl‐p‐toluidine. Frontal sections for trabecular bone were obtained from the distal femora at a thickness of 15 mm with Leica SM2500E microtome (Leica Microsystems). Trabecular sections from the proximal tibiae were either performed modified Goldner's trichrome or tartrate‐resistant acid phosphatase (TRAP) staining for analysis of static parameters or left unstained for collection of fluorochrome‐based data. Bone static histomorphometric analyses for osteoblast surface (Ob.S/BS), osteoclast surface (Oc. S/BS), osteoblast number (Ob.N/B.Pm), and osteoclast number (Oc.N/B.Pm) (Marie & Kassem, 2011) and bone dynamic histomorphometric analyses for mineral apposition rate (MAR), bone formation rate (BFR/BS), and mineralizing surface (MS/BS) were made using professional image analysis software (ImageJ, NIH, Bethesda, MD, USA) under fluorescence microscope (Leica image analysis system, Q500MC). The bone histomorphometric parameters were calculated and expressed according to standardized nomenclature for bone histomorphometry (Parfitt *et al*., 1987).

### Immunohistochemistry

The femur was fixed with 4% buffered formalin and embedded with O.C.T. after decalcification with 10% EDTA. The frozen frontal sections (5 μm thickness) were cut in a freezing cryostat at −20 °C. The sections were air‐dried at room temperature, fixed in ice‐cold acetone for 10 min, permeabilized with 0.1% Triton X‐100 at room temperature for 20 min, and blocked in 5% donkey serum in PBS. The sections were then incubated overnight at 4 °C with rat/mouse monoclonal antibody to ALP (1:500 dilutions; Abcam), PLEKHO1 (1: 100; Santa Cruz Biotechnology), and phosphorylated Smad1/5 (1:500; Cell Signaling Technology, Inc.), respectively. Following three washes in PBS, the sections were incubated with Alexa Fluor 555‐conjugated donkey anti‐mouse, or anti‐goat, or anti‐rabbit IgG (1:1000 dilution; Invitrogen) for 1 h. Negative control experiments were done by omitting the primary antibodies. The sections were mounted with the medium containing DAPI (Sigma). The sections were examined under a Nikon 80i Fluorescent Microscope or a Leica TCS SP8 Confocal Laser Scanning Microscope_ENREF_28 (Wang *et al*., 2013). For quantitative analysis, the number of PLEKHO1 and ALP co‐positive (PLEKHO1+ & ALP+) cells, pSmad1/5 and ALP co‐positive (p‐Smad1/5+ & ALP+) cells, and ALP+ cells at one sections was calculated blindly by three researchers in this study and the average value was used. Accordingly, the ratio of p‐Smad1/5+ to ALP+ cells among the ALP+ cells (pSmad1/5+ & ALP+ cell num./ALP+ cell num.) was calculated.

### Alkaline phosphatase staining

Alkaline phosphatase staining was monitored using a fast violet B salt kit (Sigma), as reported. Briefly, one fast violet B salt capsule was dissolved in 48 mL of distilled water and 2 mL of naphthol AS‐MX phosphate alkaline solution. Cells were fixed by immersion in a citrate‐buffered acetone solution (two parts citrate and three parts acetone) for 30 s and rinsed in deionized water for 45 s. The samples were then placed in an alkaline phosphatase stain for 30 min. The whole procedure was protected from light. After 2 min of rinsing in deionized water, slides were treated with Mayer's hematoxylin solution for 10 min.

### Alizarin red staining

Cells were fixed in 70% ice‐cold ethanol for 1 h and rinsed with double‐distilled H_2_O. Cells were stained with 40 mm Alizarin Red S (Sigma), pH 4.0, for 15 min with gentle agitation. Cells were rinsed five times with double‐distilled H2O and then rinsed for 15 min with 1× PBS while gently agitating.

### Statistical analysis

All the variables were expressed as mean ± standard deviation. One‐way ANOVA with LSD's post hoc test or two‐way ANOVA with a Turkey's multiple comparisons test was performed to determine intergroup differences in the study variables. A statistical software program (graphpad prism version 6.02, La Jolla, CA, USA) was used and *P* < 0.05 was considered to be statistically significant.

## Funding

This study was supported by the Ministry of Science and Technology of China (2013ZX09301307 to A.L.), the Hong Kong General Research Fund (HKBU479111 to G.Z., HKBU478312 to G.Z., HKBU262913 to G.Z., HKBU261113 to A.L., CUHK 14108816 to B.Z., CUHK14112915 to B.Z. and CUHK489213 to B.Z.), the Natural Science Foundation Council of China (81272045 to G.Z. 81272045 to B.G., 81401833 to B.G. and 81470072 to X.H.), the Research Grants Council & Natural Science Foundation Council of China (N_HKBU435/12 to G.Z.), the Croucher Foundation (Gnt#CAS14BU/CAS14201 to G.Z.), the Interdisciplinary Research Matching Scheme (IRMS) of Hong Kong Baptist University (RC‐IRMS/12‐13/02 to A.L. and RC‐IRMS/13‐14/02 to G.Z.), the Hong Kong Baptist University Strategic Development Fund (SDF) (SDF13‐1209‐P01 to A.L.), the Hong Kong Research Grants Council (RGC) Early Career Scheme (ECS) (489213 to G.Z.), the Inter‐institutional Collaborative Research Scheme of Hong Kong Baptist University (RC‐ICRS/14‐15/01 to G.Z.), the Faculty Research Grant of Hong Kong Baptist University (FRG1/13‐14/024 to G.Z. FRG2/12‐13/027 to G.Z. and FRG2/14‐15/010 to G.Z.), and the China Academy of Chinese Medical Sciences (Z0252 and Z0293 to A.L.).

## Author contributions

J.L., C.L., B.G. and X.W. performed the majority of the experiments, analyzed data and prepared the manuscript. D.L. and Z.Z helped with the *in vivo* study. K.Z. and X.H. maintained the mice. L.D. prepared the delivery system. Cw.Lu., S.P. and X.P. collected human bone specimens. G.Z., A.L., and B.Z. supervised the project and wrote most of the manuscript.

## Competing interests

The authors declare no competing financial interests.

## Supporting information


**Fig. S1** Age‐related changes of bone formation and the PLEKHO1 expression and Smad1‐dependent BMP signaling within osteoblast in aging rodents.
**Fig. S2** No obvious effect of PLEKHO1 deletion on CK2‐BRIa interaction and BRIa activation in primary osteoblasts (a) Levels of CK2, BRIa and p‐BRIa in primary osteoblasts isolated from WT or PLEKHO1 knockout mice, as determined by immunoblot analysis. (b) Interaction between CK2 and BRIa in primary osteoblasts isolated from WT or PLEKHO1 knockout mice, as determined by immunoprecipitation followed with immunoblot analysis.
**Fig. S3** Characterization of osteoblast‐specific *Plekho1* knockout mice.
**Fig. S4** Attenuated decrease in bone formation in female and male osteoblast‐specific *Plekho1* knockout mice during aging.
**Fig. S5** Characterization of osteoblast‐specific *Smad1* knock‐in mice.
**Fig. S6** Characterization of osteoblast‐specific *Plekho1* knock‐in mice and osteoblast‐specific *Smad1 and Plekho1* double knock‐in mice.
**Fig. S7** Enhanced bone formation and increased bone mass by silencing *Plekho1* within osteoblasts in aging male rats.
**Table S1** T‐score calculated from BMD measurement at L2–L4 by Dual‐energy X‐ray absorptiometry (DXA) in the fractured patients.
**Table S2** Raw data of the MicroCT and bone histomorphometry analysis in Fig. 3.
