# Supplementary material for: Increased PLEKHO1 within osteoblasts suppresses Smad‐dependent BMP signaling to inhibit bone formation during aging
Source: Aging Cell. 2017 Jan 13;16(2):360–76. doi: 10.1111/acel.12566 (PMC5334543; doi:10.1111/acel.12566)
Supplement: Supplementary file 1 — Fig. S1 Age‐related changes of bone formation and the PLEKHO1 expression and Smad1‐dependent BMP signaling within osteoblast in aging rodents. Fig. S2 No obvious effect of PLEKHO1 deletion on CK2‐BRIa interaction and BRIa activation in primary osteoblasts (a) Levels of CK2, BRIa and p‐BRIa in primary osteoblasts isolated from WT or PLEKHO1 knockout mice, as determined by immunoblot analysis. (b) Interaction between CK2 and BRIa in primary osteoblasts isolated from WT or PLEKHO1 knockout mice, as determined by immunoprecipitation followed with immunoblot analysis. Fig. S3 Characterization of osteoblast‐specific Plekho1 knockout mice. Fig. S4 Attenuated decrease in bone formation in female and male osteoblast‐specific Plekho1 knockout mice during aging. Fig. S5 Characterization of osteoblast‐specific Smad1 knock‐in mice. Fig. S6 Characterization of osteoblast‐specific Plekho1 knock‐in mice and osteoblast‐specific Smad1 and Plekho1 double knock‐in mice. Fig. S7 Enhanced bone formation and increased bone mass by silencing Plekho1 within osteoblasts in aging male rats. Table S1 T‐score calculated from BMD measurement at L2–L4 by Dual‐energy X‐ray absorptiometry (DXA) in the fractured patients. Table S2 Raw data of the MicroCT and bone histomorphometry analysis in Fig. 3. [file ACEL-16-360-s001.pdf]

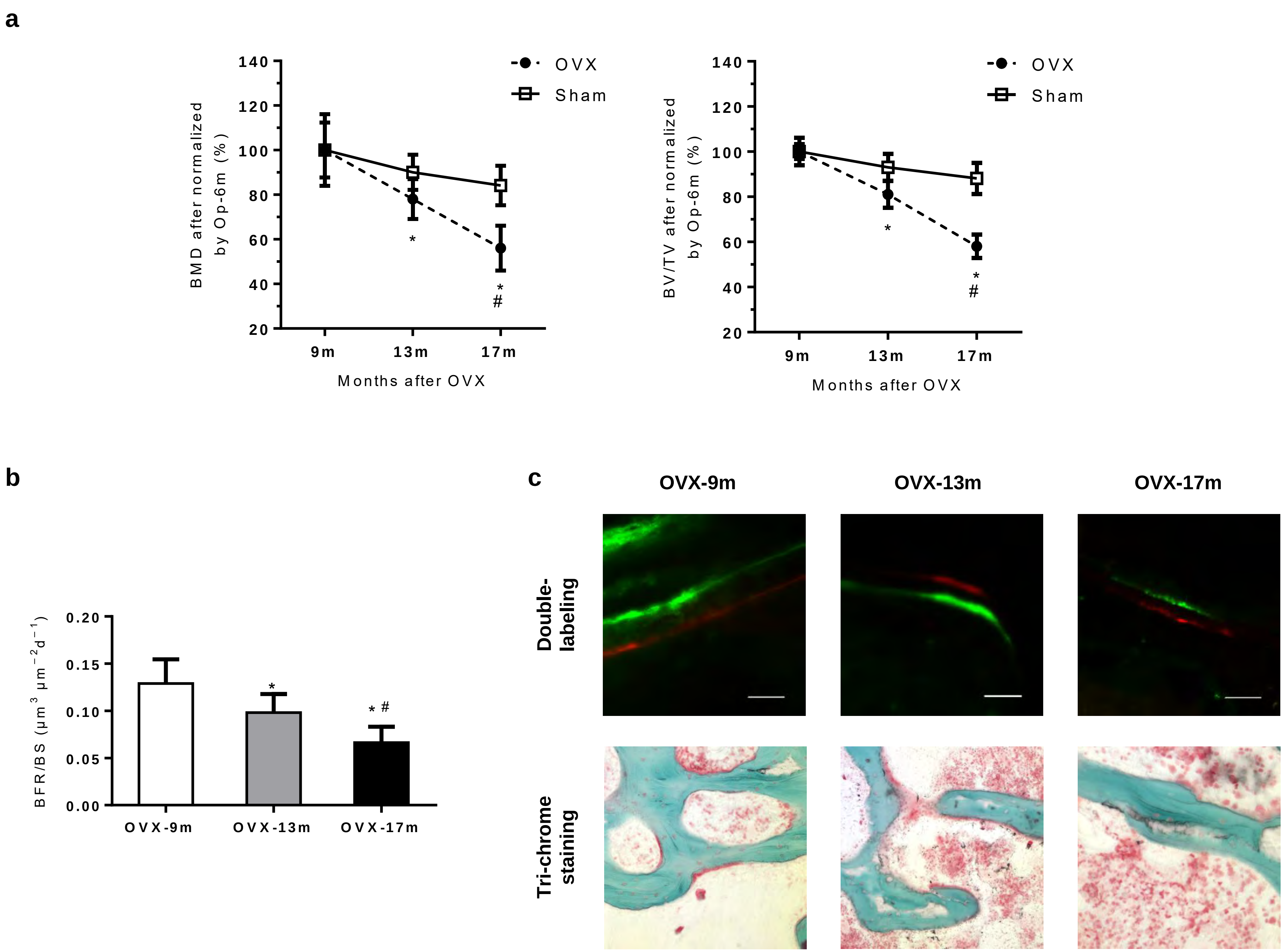
a
140
120
100
80
60
40
20
BMD after normalized by Op-6m (%)
OVX
Sham
9m
13m
17m
Months after OVX
*
#
BV/TV after normalized by Op-6m (%)
b
BFR/BS (μm³ μm⁻² d⁻¹)
0.20
0.15
0.10
0.05
0.00
OVX-9m
OVX-13m
OVX-17m
c
OVX-9m
OVX-13m
OVX-17m
Double-labeling
Tri-chrome staining

d

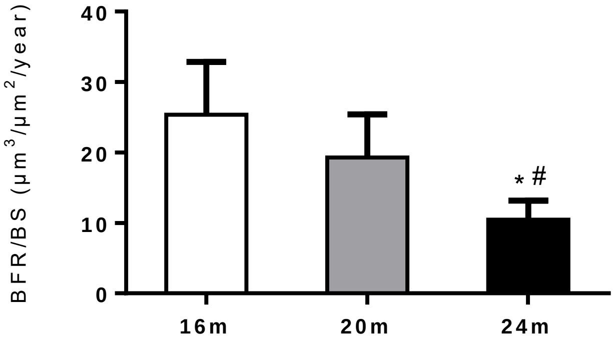

BFR/BS (μm³/μm²/year)
40
30
20
10
0
* #
16m
20m
24m

e

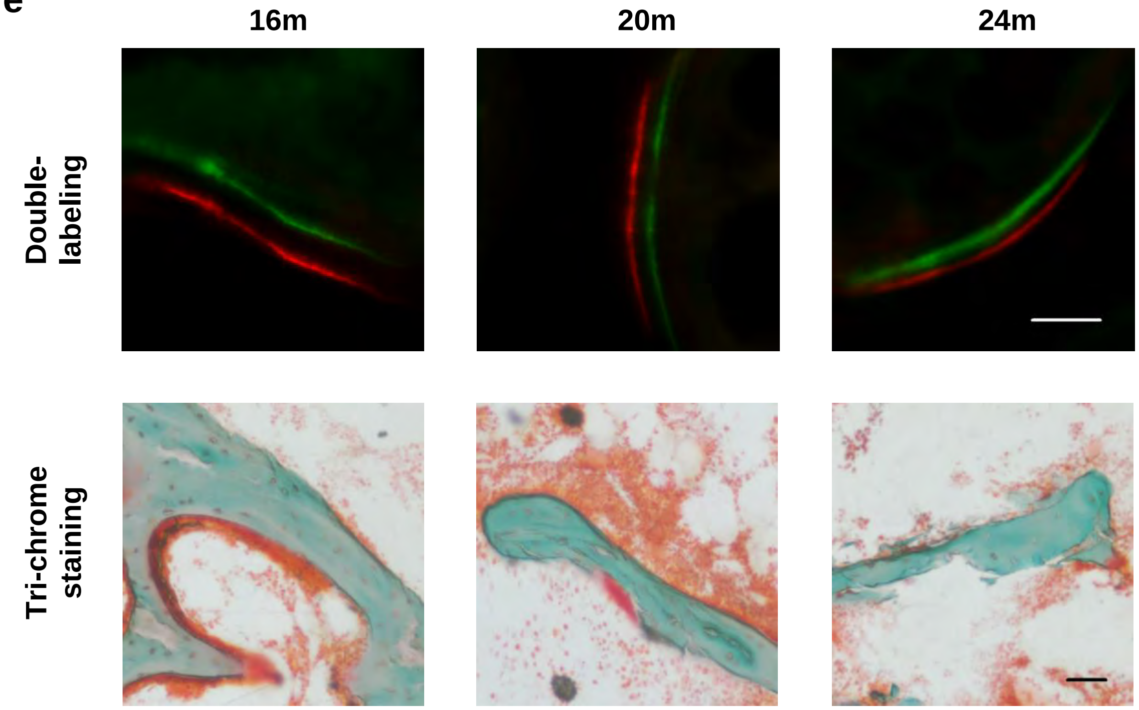

16m
20m
24m
Double-labeling
Tri-chrome staining

f

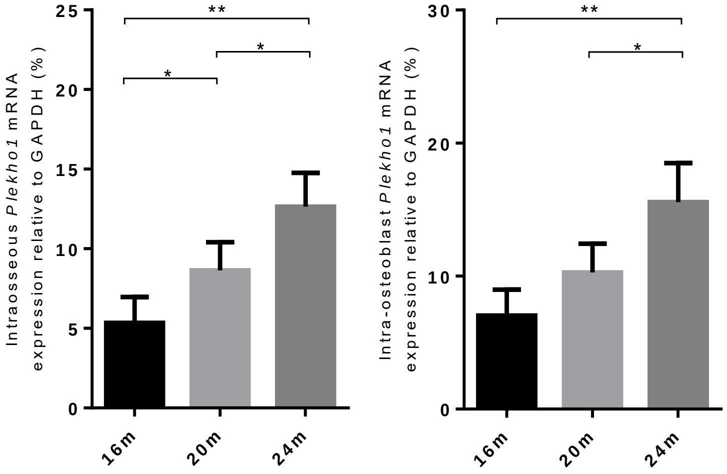

Intraosseous Plekho1 mRNA expression relative to GAPDH (%)
25
20
15
10
5
0
**
*
*
16m
20m
24m
Intra-osteoblast Plekho1 mRNA expression relative to GAPDH (%)
30
20
10
0
**
*
16m
20m
24m

g

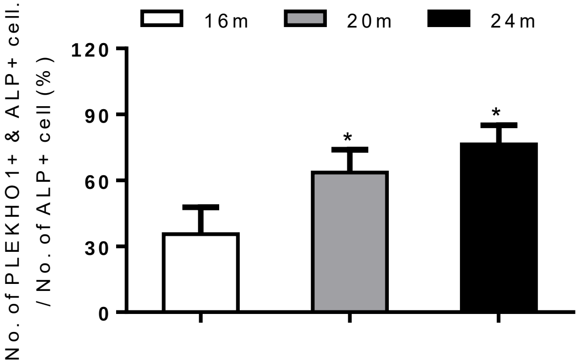

16m
20m
24m
No. of PLEKHO1+ & ALP+ cell. / No. of ALP+ cell (%)
120
90
60
30
0
*
*

h

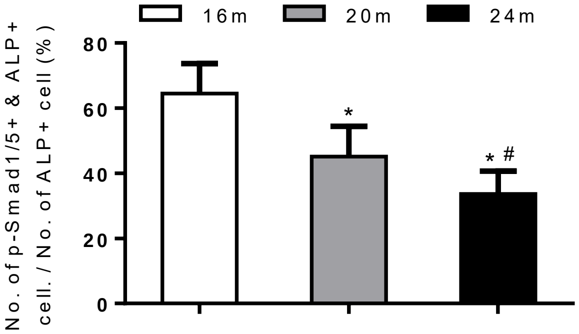

16m
20m
24m
No. of p-Smad1/5+ & ALP+ cell. / No. of ALP+ cell (%)
80
60
40
20
0
*
* #

Supplementary Figure 1 Age-related changes of bone formation and the PLEKHO1 expression and Smad1-dependent BMP signaling within osteoblast in aging rodents. (a) Quantitative data of *in vivo* microCT analysis at the distal femora from ovariectomized rats and age-matched sham-operated controls. **(b)** The age-related changes of bone formation rate (BFR/BS) at distal femur from the aging ovariectomized (OVX) rats. **(c)** The representative micrographs of newly mineralized bone assessed by xylenol (red) and calcein (green) labeling (upper panel) and osteoid indicated by Masson's trichrome staining (lower panel) at distal femur from aging OVX rats at 9 (OVX-9m), 13 (OVX-13m) and 17 (OVX-17m) months after ovariectomy. Scale bars = 10 μm. **(d)** The age-related changes of bone formation rate (BFR/BS) at distal femur from the aging male rats. **(e)** The representative micrographs of newly mineralized bone assessed by xylenol (red) and calcein (green) labeling (upper panel) and osteoid indicated by Masson's trichrome staining (lower panel) at distal femur from aging male rats at 16, 20 and 24 months of age. Scale bars = 10 μm. **(f)** The age-related changes of *Plekho1* mRNA levels in whole bone tissue (left) and osteoblast (right) from aging male rats. **(g)** The age-related changes of the ratio of PLEKHO1 and ALP co-positive cells (PLEKHO1+ & ALP+) among the ALP+ cells at distal femur from aging male rats calculated by immunofluorescence analysis. **(h)** The age-related changes of the ratio of pSmad1/5 and ALP co-positive cells (p-Smad1/5+ & ALP+) among the ALP+ cells at distal femur from aging male rats by immunofluorescence analysis. **Note**: Rat *Gapdh* mRNA are used as the internal controls. In (a), (c), (f) and (g) * $P < 0.05$ when compared to the OVX-9m / 16m group. # $P < 0.05$ when compared to the OVX-17m / 20 m group. In (d), * $P<0.05$, ** $P<0.01$. One-way analysis of variance (ANOVA) with a *post-hoc* test was performed.

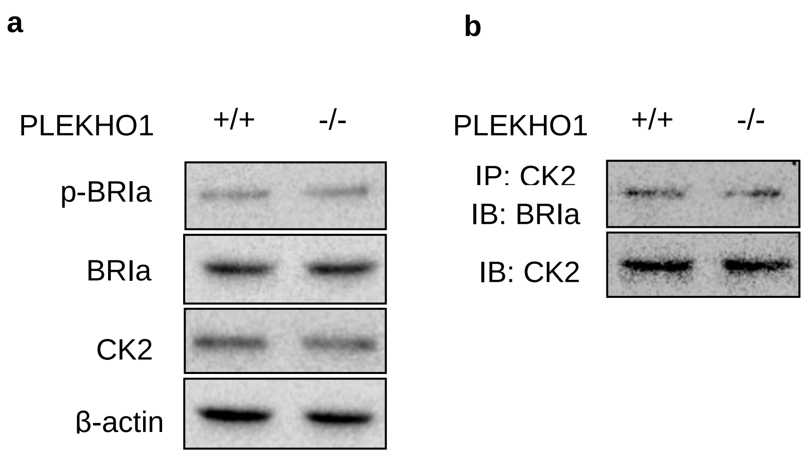

Supplementary Figure 2 No obvious effect of PLEKHO1 deletion on CK2-BRIa interaction and BRIa activation in primary osteoblasts (a) Levels of CK2, BRIa and p-BRIa in primary osteoblasts isolated from WT or PLEKHO1 knockout mice, as determined by immunoblot analysis. **(b)** Interaction between CK2 and BRIa in primary osteoblasts isolated from WT or PLEKHO1 knockout mice, as determined by immunoprecipitation followed with immunoblot analysis.

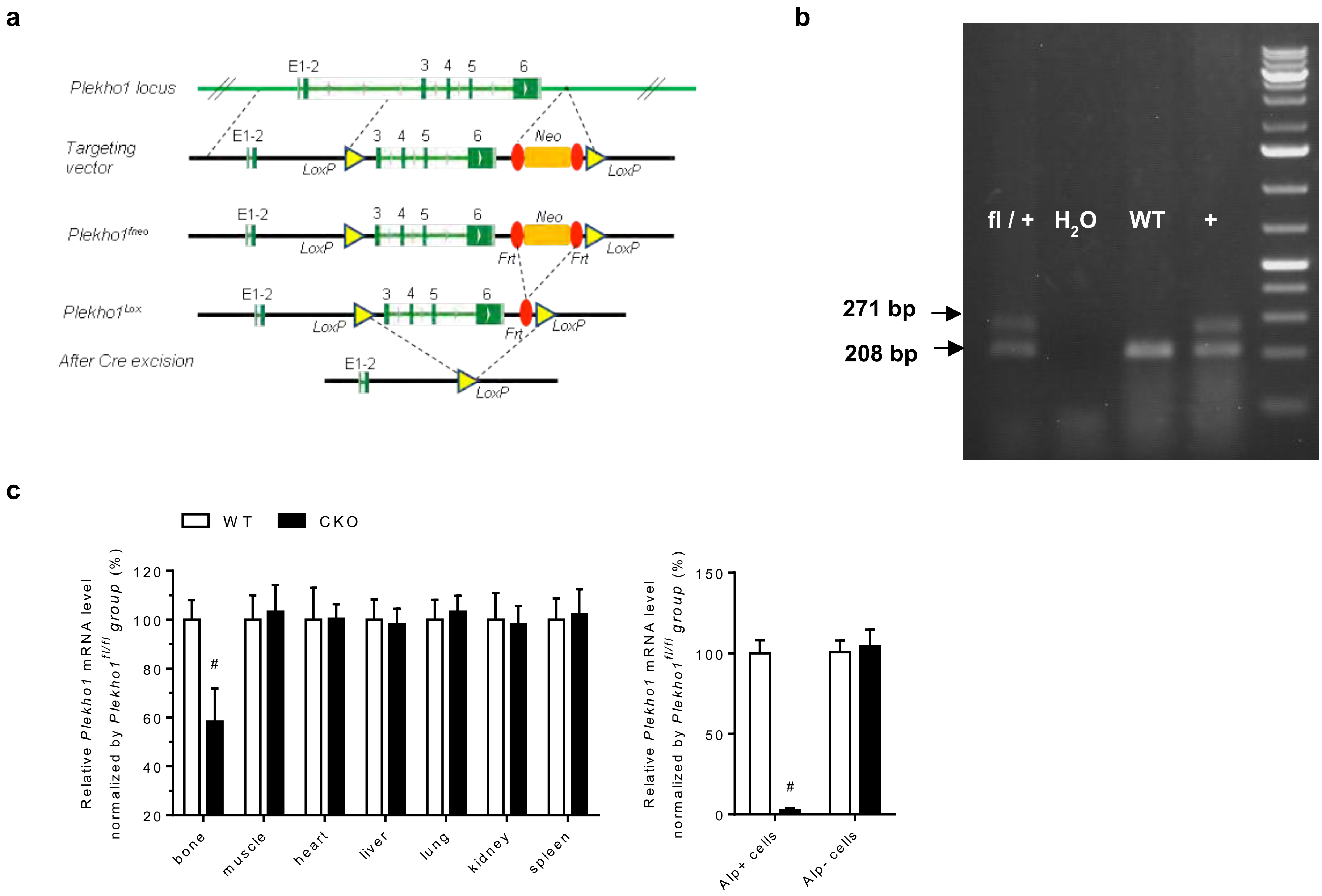

a
Plekho1 locus
E1-2
3 4 5 6
Targeting vector
LoxP
Neo
Plekho1^fneo
Frt
Plekho1^Lox
After Cre excision
b
fl / +
H2O
WT
+
271 bp
208 bp
c
WT
CKO
Relative Plekho1 mRNA level normalized by Plekho1^fl/fl group (%)
120
100
80
60
40
20
#
bone
muscle
heart
liver
lung
kidney
spleen
150
100
50
0
Alp+ cells
Alp- cells

Supplementary Figure 3 Characterization of osteoblast-specific *Plekho1* knockout mice. (a) Schematic diagram for development strategy to generate *Plekho1*$^{floxp/-}$ mice. **(b)** Genotyping analysis of the *Plekho1*$^{floxp/-}$ mice. **(c)** The *Plekho1* mRNA levels in bone versus non-bone tissues (left) and osteoblasts (OBs) versus non-osteoblasts (Non-OBs) (left) from *Osx;Plekho1*$^{fl/fl}$ (CKO) and *Plekho1*$^{fl/fl}$ (WT) mice.

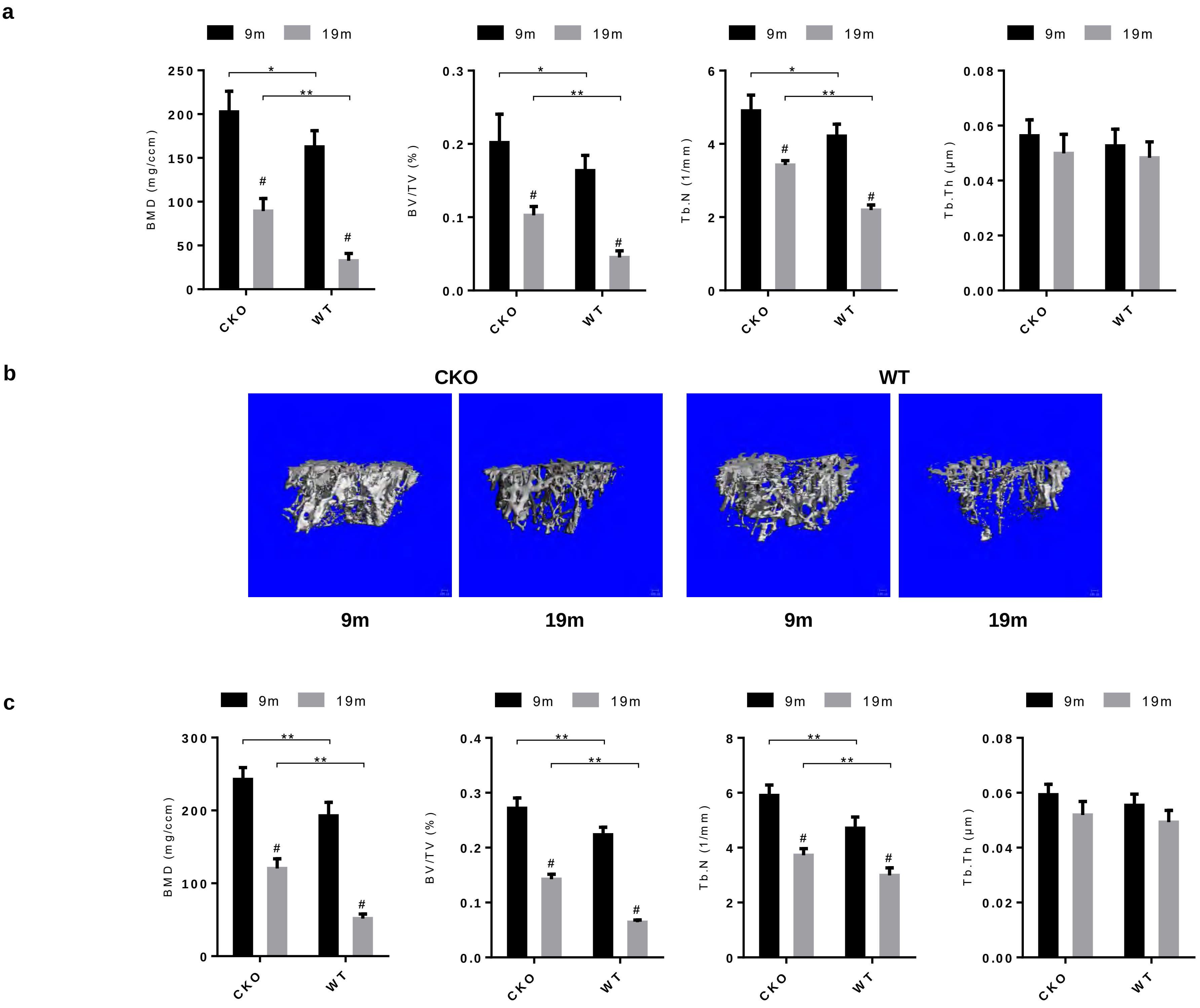
a
9m
19m
BMD (mg/ccm)
BV/TV (%)
Tb.N (1/mm)
Tb.Th (μm)
CKO
WT
*
**
#
b
CKO
WT
9m
19m
9m
19m
c
9m
19m
BMD (mg/ccm)
BV/TV (%)
Tb.N (1/mm)
Tb.Th (μm)
CKO
WT
**
#

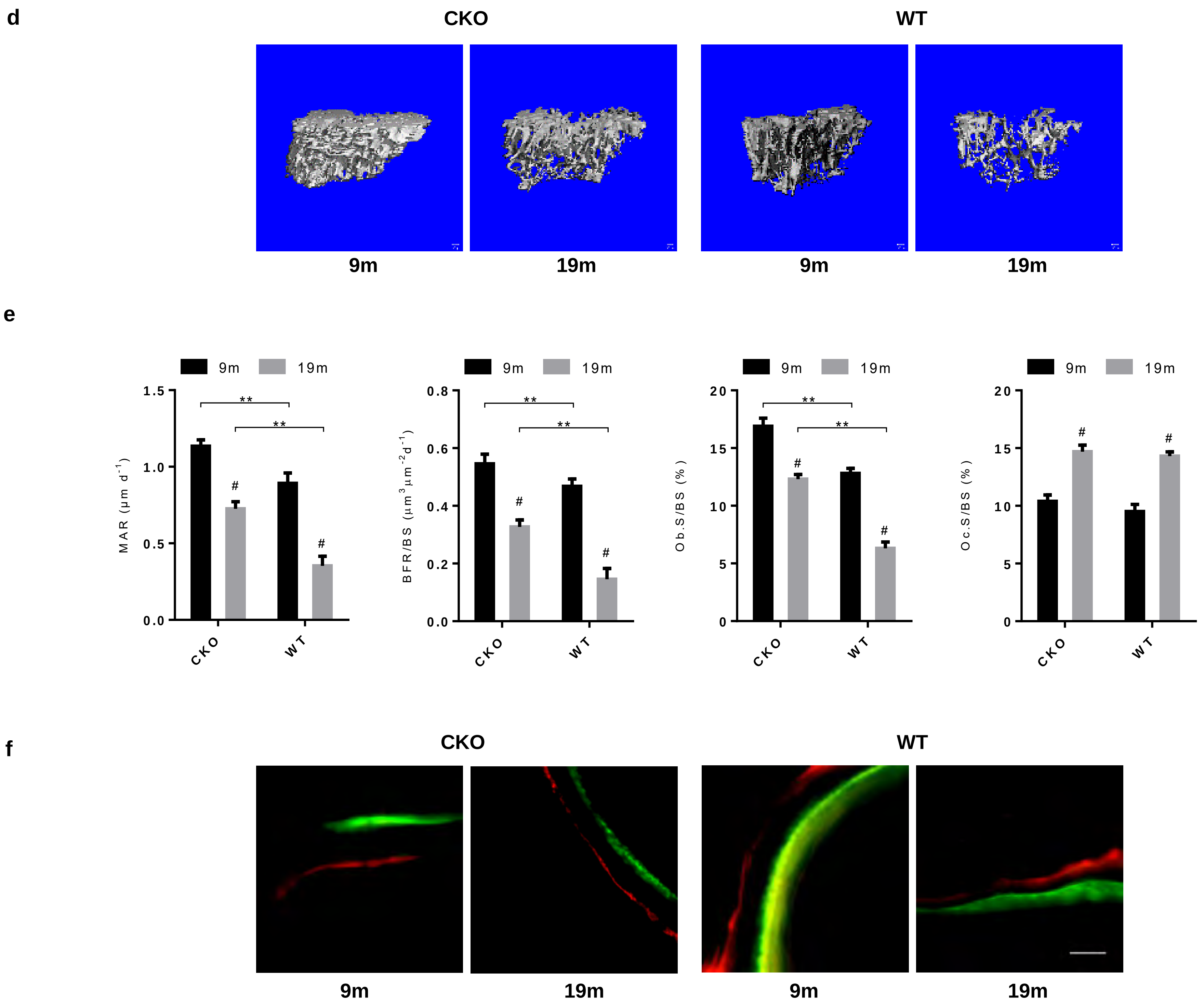

d
CKO
WT
9m
19m
9m
19m
e
9m
19m
MAR (μm d⁻¹)
1.5
1.0
0.5
0.0
**
**
#
#
CKO
WT
BFR/BS (μm³μm⁻²d⁻¹)
0.8
0.6
0.4
0.2
0.0
Ob.S/BS (%)
20
15
10
5
0
Oc.S/BS (%)
f
CKO
WT
9m
19m
9m
19m

Supplementary Figure 4 Attenuated decrease in bone formation in female and male osteoblast-specific *Plekho1* knockout mice during aging. **(a)** The age-related changes of micro-CT parameters at proximal tibiae from the female *Osx;Plekho1*$^{fl/fl}$ (CKO) and *Plekho1*$^{fl/fl}$ (WT) mice with sham operation. **(b)** Representative 3-D microCT images of trabecular micro-architecture at proximal tibiae from female CKO and WT mice with sham operation during aging. Scale bar = 100 μm. **(c)** The age-related changes of micro-CT parameters at proximal tibiae from the male CKO and WT mice. **(d)** Representative 3-D microCT images of trabecular micro-architecture at proximal tibiae from male CKO and WT mice during aging. Scale bar = 100 μm **(e)** The age-related changes of bone histomorphometric parameters at proximal tibiae from male CKO and WT mice. **(f)** Representative micrographs of newly mineralized bone assessed by both xylenol (red) and calcein (green) labeling at proximal tibiae from male CKO and WT mice during aging. Scale bar = 5 μm. **Note**: * *P*<0.05, ** *P*<0.01. # *P*<0.05 vs. 9m. Two-way analysis of variance (ANOVA) with a Turkey's multiple comparisons test was performed.

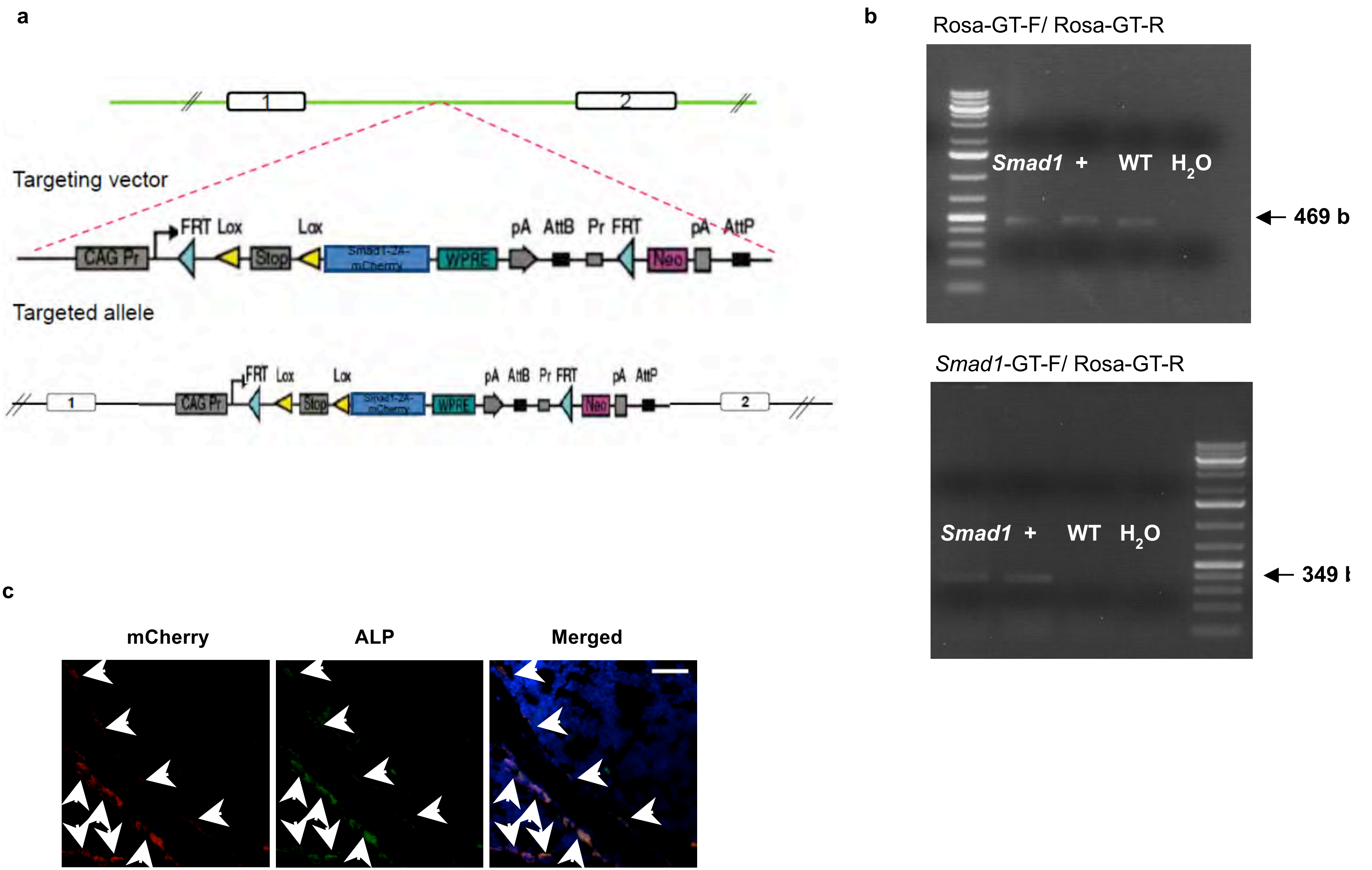

a
Targeting vector
Targeted allele
CAG Pr
FRT
Lox
Stop
Lox
Smad1-2A-mCherry
WPRE
pA
AttB
Pr
FRT
Neo
pA
AttP
1
2
b
Rosa-GT-F/ Rosa-GT-R
Smad1 + WT H_2O
469 b
Smad1-GT-F/ Rosa-GT-R
Smad1 + WT H_2O
349
c
mCherry
ALP
Merged

d

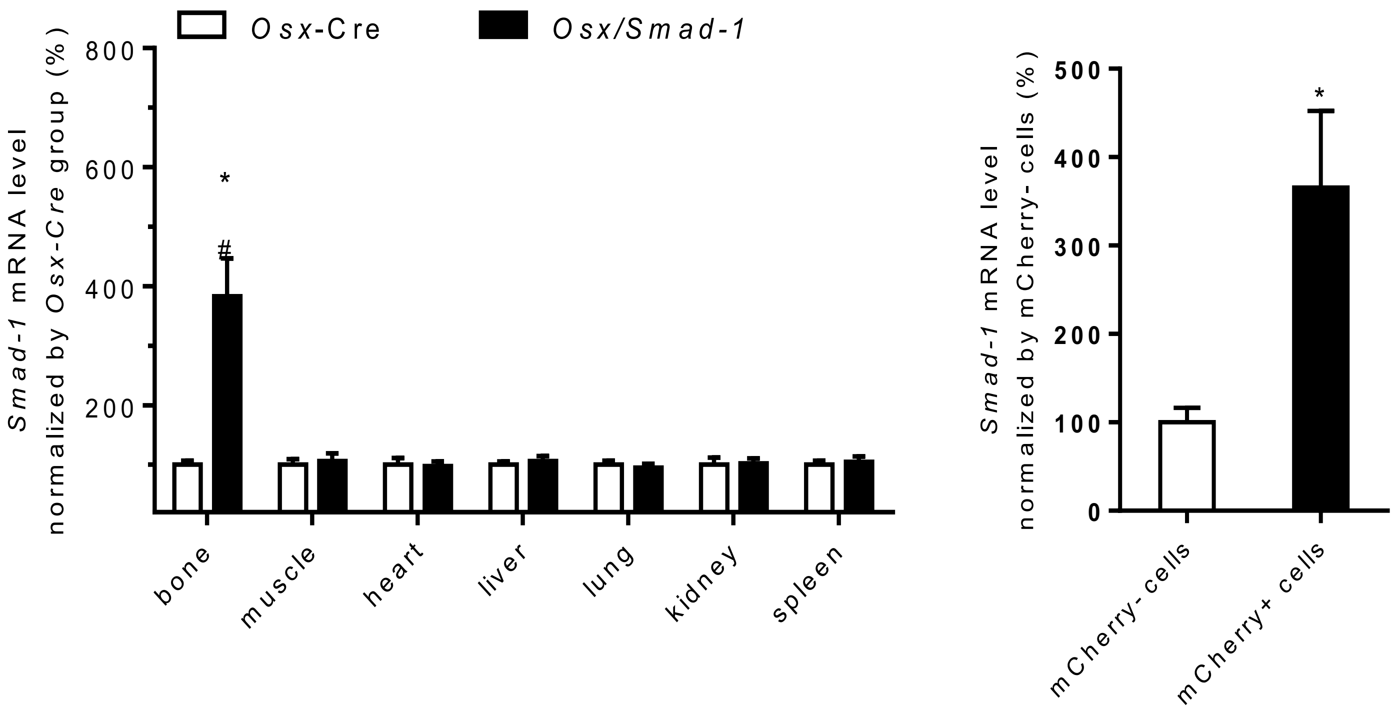

Supplementary Figure 5 Characterization of osteoblast-specific *Smad1* knock-in mice. (a) Schematic diagram for development strategy to generate *ROSA26-PCAG-STOPfl-Smad1-mCherry* knock-in mice. **(b)** Genotyping analysis of the *ROSA26-PCAG-STOPfl-Smad1-mCherry* knock-in mice. **(c)** The representative fluorescence micrographs showing the co-localization of Smad1 (mCherry, red) + and ALP+ (green) cells at tibiae cyosections from *Osx/Smad1* mice. Arrows indicate Smad1 and ALP co-positive cells. Scale bar: 100 μm. **(d)** The *Smad1* mRNA levels in bone versus non-bone tissues from *Osx/Smad1* and *Osx-Cre* mice (left) and mCherry+ cells (OBs) versus mCherry- cells (Non-OBs) (right) from *Osx/Smad1* mice. The mCherry+ cells were isolated from bone marrow cells by fluorescence activated cell sorting. $^{\#}P$<0.05.

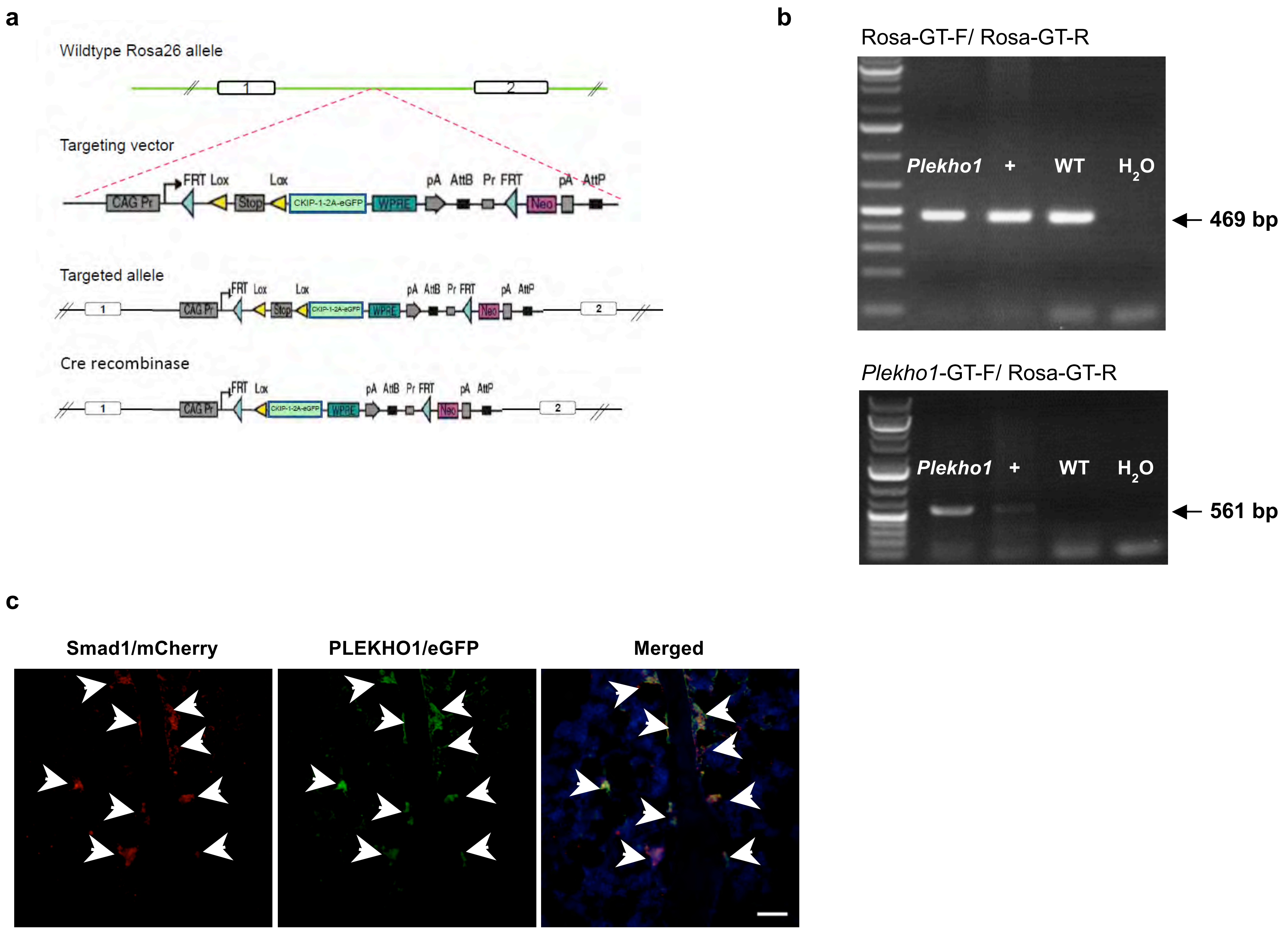

a
Wildtype Rosa26 allele
1
2
Targeting vector
CAG Pr
FRT Lox
Stop
Lox
CKIP-1-2A-eGFP
WPRE
pA AttB Pr FRT
Neo
pA AttP
Targeted allele
Cre recombinase
b
Rosa-GT-F/ Rosa-GT-R
Plekho1 + WT H2O
469 bp
Plekho1-GT-F/ Rosa-GT-R
561 bp
c
Smad1/mCherry
PLEKHO1/eGFP
Merged

d

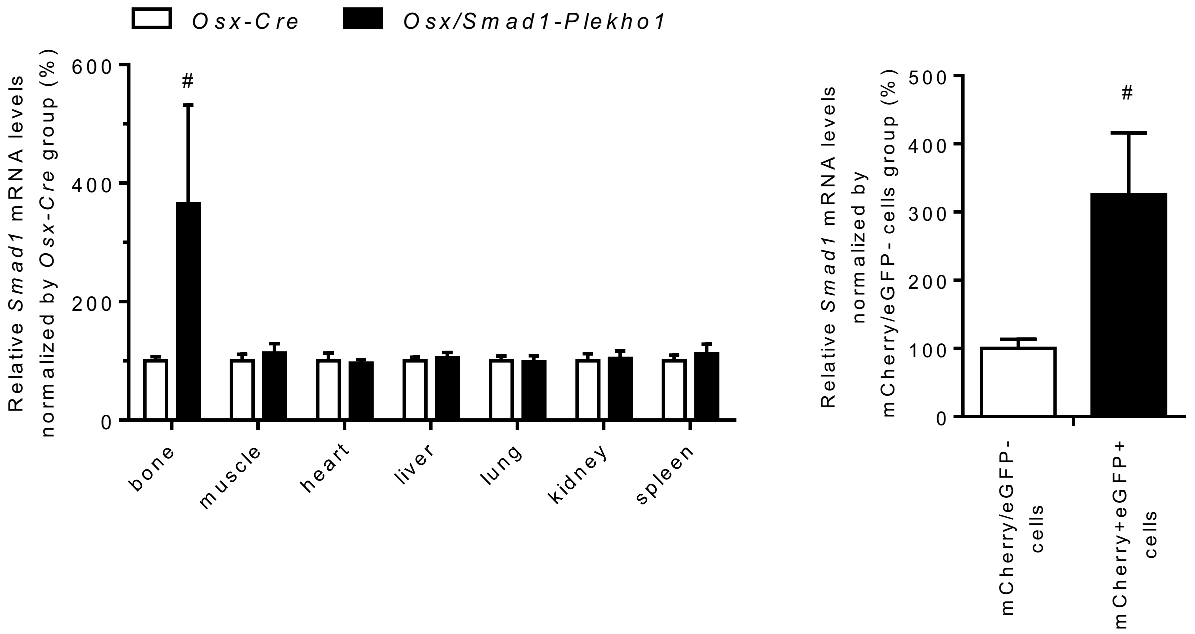

Osx-Cre
Osx/Smad1-Plekho1
Relative Smad1 mRNA levels normalized by Osx-Cre group (%)
600
400
200
0
#
bone
muscle
heart
liver
lung
kidney
spleen
Relative Smad1 mRNA levels normalized by mCherry/eGFP- cells group (%)
500
400
300
200
100
0
#
mCherry/eGFP- cells
mCherry+eGFP+ cells

e

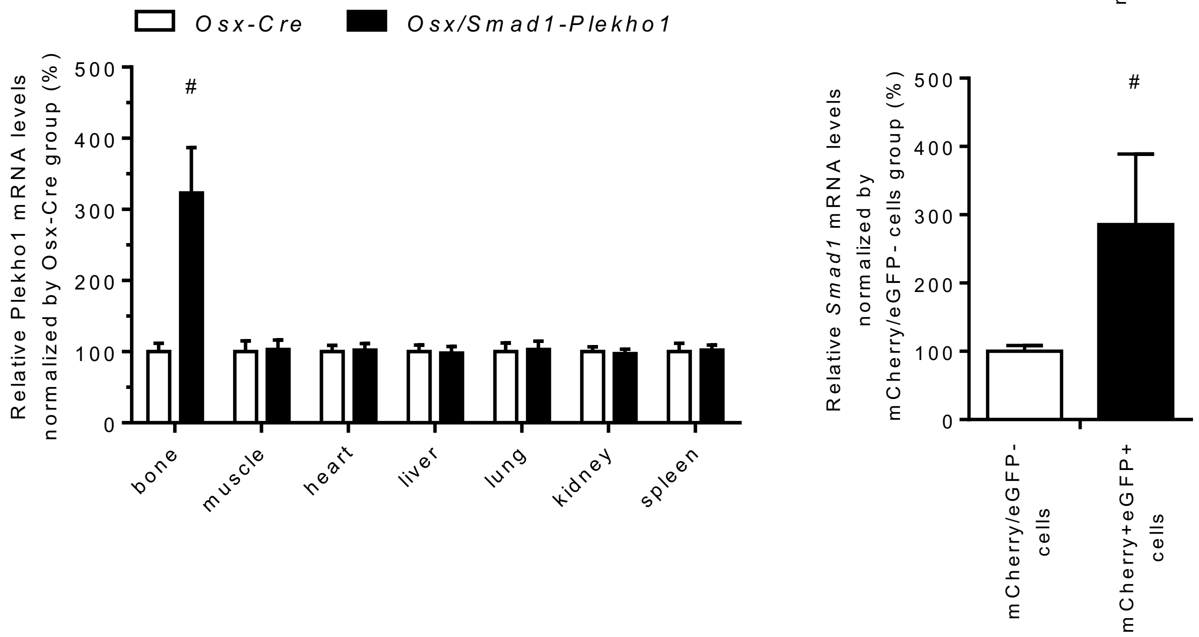

Osx-Cre
Osx/Smad1-Plekho1
Relative Plekho1 mRNA levels normalized by Osx-Cre group (%)
500
400
300
200
100
0
#
bone
muscle
heart
liver
lung
kidney
spleen
Relative Smad1 mRNA levels normalized by mCherry/eGFP- cells group (%)
500
400
300
200
100
0
#
mCherry/eGFP- cells
mCherry+eGFP+ cells

Supplementary Figure 6 Characterization of osteoblast-specific *Plekho1* knock-in mice and osteoblast-specific *Smad1 and Plekho1* double knock-in mice. (a) Schematic diagram for development strategy to generate *ROSA26-PCAG-STOPfl-Plekho1-eGFP* knock-in mice. **(b)** Genotyping analysis of the *ROSA26-PCAG-STOPfl-Plekho1-eGFP* knock-in mice. **(c)** The representative fluorescence micrographs showing the co-localization of Smad1 (mCherry, red) + and PLEKHO+ (eGFP, green) cells at tibiae cyosections from *Osx/Smad1-PLEKHO1* mice. Arrows indicate Smad1 and PLEKHO1 co-positive cells. Scale bar: 100 μm. **(d)** The *Smad1* mRNA levels in bone versus non-bone tissues from *Osx/Smad1-Plekho1* and *Osx-Cre* mice (left) and mCherry and eGFP co-positive cells versus mCherry or/and eGFP negative cells (right) from *Osx/Smad1-Plekho1* mice. **(e)** The *Plekho11* mRNA levels in bone versus non-bone tissues from *Osx/Smad1-Plekho1* and *Osx-Cre* mice (left) and mCherry and eGFP co-positive cells versus mCherry or/and eGFP negative cells (right) from *Osx/Smad1-Plekho1* mice. The mCherry and eGFP co-positive cells were isolated from bone marrow cells by fluorescence activated cell sorting. $^{\#}P$<0.05.

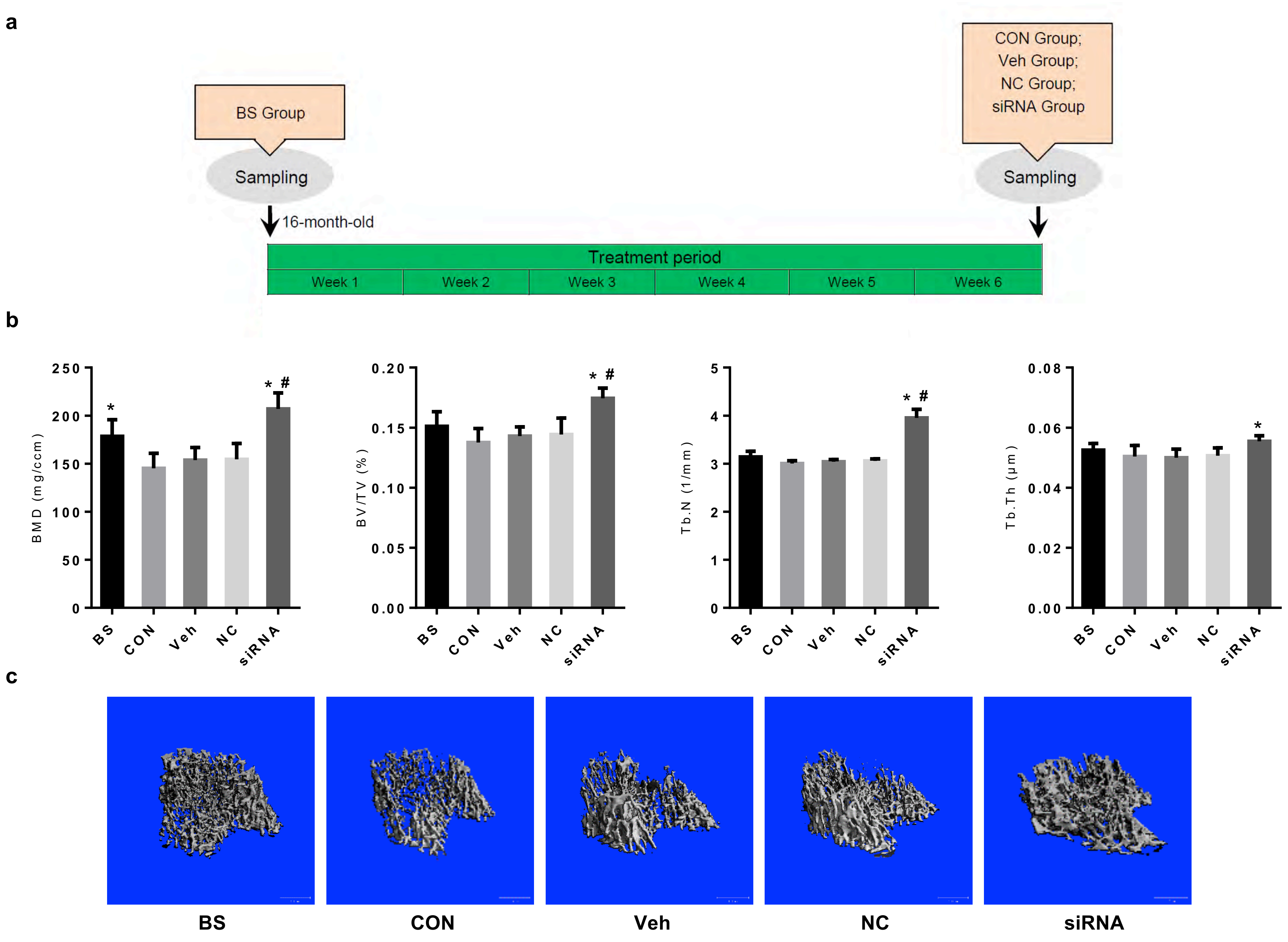
a
BS Group
Sampling
16-month-old
CON Group;
Veh Group;
NC Group;
siRNA Group
Sampling
Treatment period
Week 1
Week 2
Week 3
Week 4
Week 5
Week 6
b
BMD (mg/ccm)
BV/TV (%)
Tb.N (1/mm)
Tb.Th (μm)
BS
CON
Veh
NC
siRNA
c
BS
CON
Veh
NC
siRNA

d

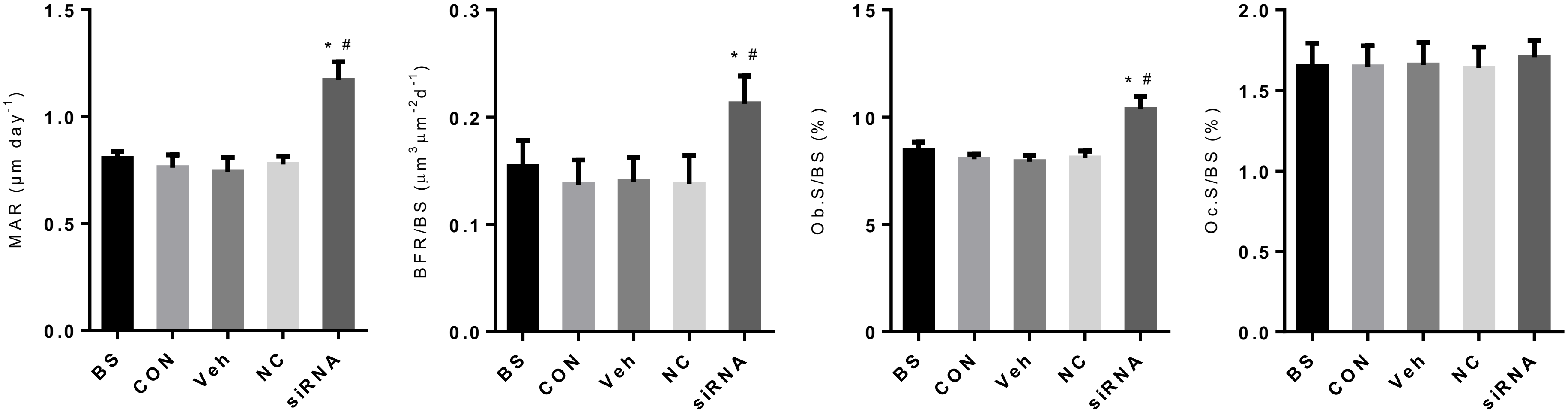

e

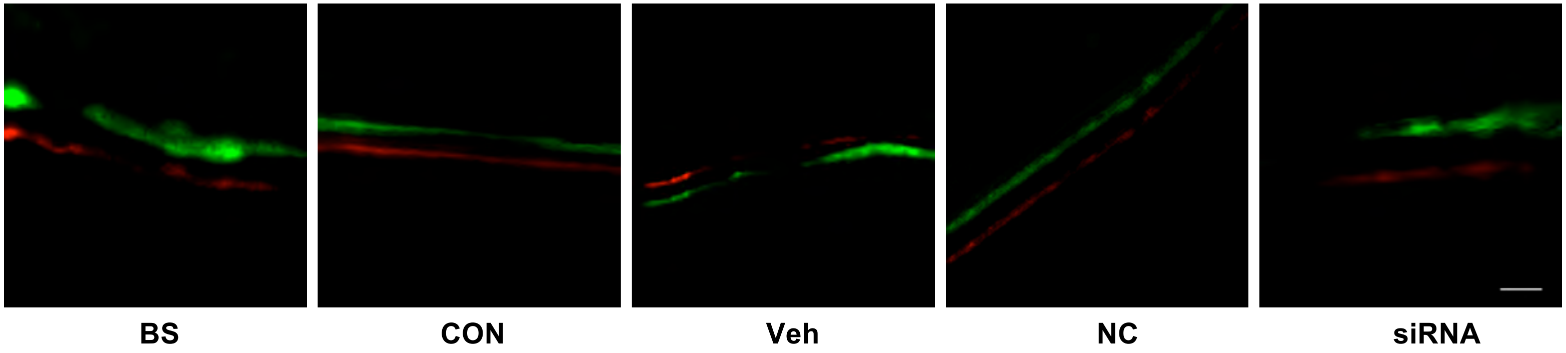

Supplementary Figure 7 Enhanced bone formation and increased bone mass by silencing *Plekho1* within osteoblasts in aging male rats. **(a)** A schematic diagram illustrating the experimental design. **(b)** The quantitative data of micro-CT parameters at proximal tibiae in the indicated group. **(c)** The representative 3-D microCT images of trabecular micro-architecture at proximal tibiae in each group. Scale bar = 1 mm **(d)** The quantitative data of bone histomorphometric parameters at proximal tibiae in the indicated group. **(e)** The representative micrographs of newly mineralized bone assessed by both xylenol (red) and calcein (green) labeling at proximal tibiae in the indicated group. Scal bar = 10 μm. **Note:** All data are mean ± sd. * $P<0.05$ vs. either CON, NC or Veh Group. # $P<0.05$ vs. BS Group. One-way analysis of variance (ANOVA) with a *post-hoc* test was performed.

Supplementary Table 1 T-score calculated from BMD measurement at L2~L4 by Dual-energy X-ray absorptiometry (DXA) in the fractured patients.

Gender	Age	Number	T score at L2~L4
Women	60~69	12	-1.83±0.52
	70~79	8	-2.29±0.38
	80~89	9	-2.58±0.41
Men	60~69	8	-1.48±0.43
	70~79	7	-1.79±0.55
	80~89	6	-2.29±0.51

Supplementary Table 2

Raw data of the MicroCT and bone histomorphometry analysis in Figure 3

Parameter		CKO-9m	CKO-19m	WT-9m	WT-19m
BMD	Mean	157.52	62.18	97.11	19.04
	SD	28.41	8.26	12.02	4.74
BV/TV	Mean	0.17	0.07	0.11	0.03
	SD	0.034	0.008	0.016	0.002
Tb.N	Mean	4.13	3.15	3.24	1.91
	SD	0.43	0.20	0.47	0.45
Tb.Th	Mean	0.056	0.047	0.053	0.042
	SD	0.003	0.005	0.005	0.002
MAR	Mean	0.923	0.620	0.623	0.311
	SD	0.049	0.048	0.035	0.052
BFR	Mean	0.395	0.273	0.229	0.093
	SD	0.021	0.028	0.018	0.020
OB.S	Mean	16.00	11.65	11.06	5.78
	SD	0.50	0.43	0.35	0.44
OC.S	Mean	9.03	12.81	8.70	12.33
	SD	0.39	0.40	0.35	0.50

Raw data of the MicroCT and bone histomorphometry analysis in Figure 4

Parameter		Osx-Cre-9m	Osx-Cre-19m	Osx/Smad1-9m	Osx/Smad1-19m
BMD	Mean	101.10	21.12	169.11	63.36
	SD	12.85	4.80	17.52	11.51
BV/TV	Mean	0.12	0.04	0.17	0.08
	SD	0.015	0.006	0.017	0.009
MAR	Mean	0.629	0.350	0.937	0.687
	SD	0.021	0.019	0.039	0.035
BFR	Mean	0.238	0.100	0.408	0.292
	SD	0.022	0.018	0.031	0.026
